# WWC1/2 regulate spinogenesis and cognition in mice by stabilizing AMOT

**DOI:** 10.1038/s41419-023-06020-7

**Published:** 2023-08-01

**Authors:** Runyi Cao, Rui Zhu, Zhao Sha, Sixian Qi, Zhenxing Zhong, Fengyun Zheng, Yubin Lei, Yanfeng Tan, Yuwen Zhu, Yu Wang, Yi Wang, Fa-Xing Yu

**Affiliations:** 1 Institute of Pediatrics, Children’s Hospital of Fudan University, and the Shanghai Key Laboratory of Medical Epigenetics, The International Co-laboratory of Medical Epigenetics and Metabolism, the State Key Laboratory of Genetic Engineering, Institutes of Biomedical Sciences, Shanghai Medical College, Fudan University, Shanghai, 200032 China; 2 Department of Neurology, Children’s Hospital of Fudan University, National Children’s Medical Center, No. 399 Wanyuan Road, Shanghai, 201102 China

**Keywords:** Molecular neuroscience, Biochemistry

## Abstract

WWC1 regulates episodic learning and memory, and genetic nucleotide polymorphism of *WWC1* is associated with neurodegenerative diseases such as Alzheimer’s disease. However, the molecular mechanism through which WWC1 regulates neuronal function has not been fully elucidated. Here, we show that WWC1 and its paralogs (WWC2/3) bind directly to angiomotin (AMOT) family proteins (Motins), and recruit USP9X to deubiquitinate and stabilize Motins. Deletion of *WWC* genes in different cell types leads to reduced protein levels of Motins. In mice, neuron-specific deletion of *Wwc1* and *Wwc2* results in reduced expression of Motins and lower density of dendritic spines in the cortex and hippocampus, in association with impaired cognitive functions such as memory and learning. Interestingly, ectopic expression of AMOT partially rescues the neuronal phenotypes associated with *Wwc1/2* deletion. Thus, WWC proteins modulate spinogenesis and cognition, at least in part, by regulating the protein stability of Motins.

## Introduction

The WWC protein family, comprising WWC1 (WW and C2 domain-containing 1, also known as KIBRA for KIdney and BRAin), WWC2, and WWC3, have recently been identified as Hippo signaling pathway regulators [1–3]. Previous reports indicate that genetic perturbation of *Wwc1* in rodent neuronal cells leads to reduced synaptic plasticity, episodic learning, and memory [4–8]. Moreover, WWC1 is associated with neurological disorders, including impaired memory, depression, post-traumatic stress disorder (PTSD), and Alzheimer’s disease [7, 9–14]. Despite these findings, the precise molecular mechanism underlying the role of WWC1 in brain function remains unclear.

**Fig. 1:**
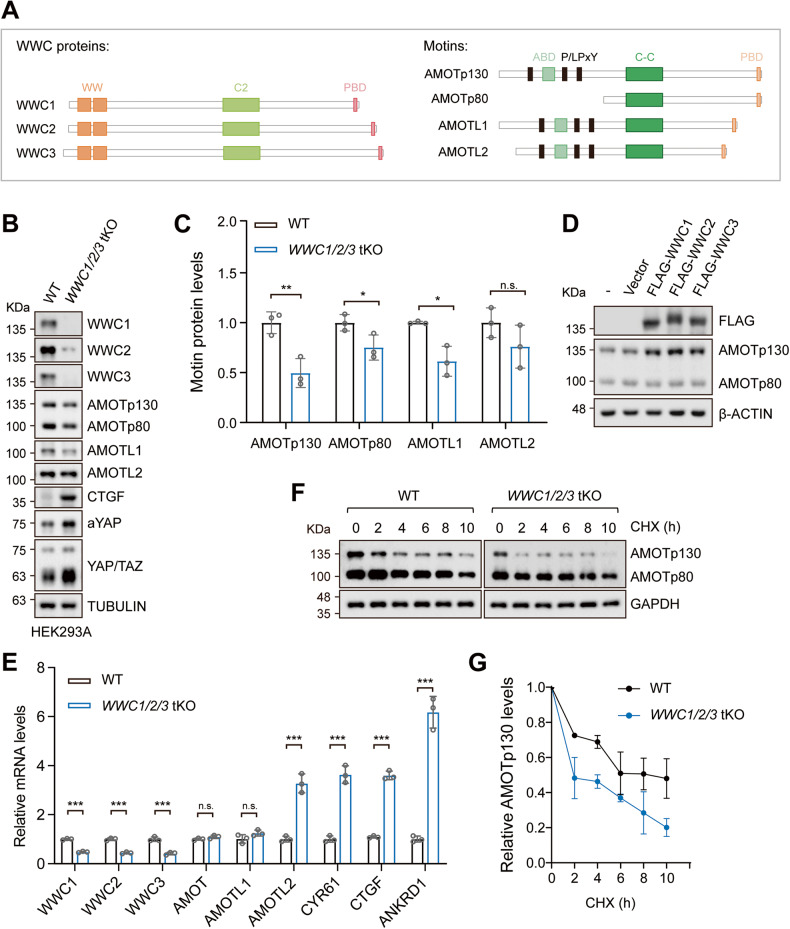
WWC1/2/3 regulate protein stability of Motins. **A** Schematic representation shows domain structures of WWC proteins and Motin family proteins. WWC1, WWC2, and WWC3 share N-terminal WW domains, an internal C2 domain and C-terminal PDZ-binding domain (PBD). Motins consist of AMOTp130, AMOTp80, AMOTL1, and AMOTL2, sharing similar C-terminal sequences. Motins contain PPxY (or LPxY) motif, F-actin binding domain (ABD), Coiled coil domain, and PBD. **B**, **C** Motins protein levels are decreased in *WWC1/2/3* tKO HEK293A cells. *WWC1/2/3* tKO represents the knockout of *WWC1*, *WWC2*, and *WWC3* genes. Protein expression was determined by immunoblotting (**B**) and quantified (**C**). Data are shown as the mean ± SD of three independent experiments. *p < 0.05, **p < 0.01, and n.s. (not significant) between indicated groups. **D** Ectopic expression of WWC1/2/3 upregulates protein levels of AMOTp130. Cell lysates from control and WWC-overexpressing HEK293A cells were subjected to immunoblotting. **E** The mRNA levels of Motins, except for *AMOTL2*, are not regulated by WWC proteins. *CTGF*, *CYR61*, *ANKRD1*, and *AMOTL2* mRNA levels were increased in *WWC1/2/3* tKO HEK293A cells. The expression levels of YAP target genes were assessed by quantitative real-time PCR. Data are shown as the mean ± SD of three independent experiments. ***p < 0.005, and n.s. (not significant) between indicated groups. **F**, **G** WWC proteins stabilize AMOTp130. Whole-cell lysates from wild-type and *WWC1/2/3* tKO HEK293A cells treated with cycloheximide (CHX; 100 mg/mL for 2 to 10 h) were collected and subjected to immunoblotting. Protein quantification is shown in **G**. Data are shown as the mean ± SD of three independent experiments.

WWC proteins contain multiple conserved domains that support protein-protein interactions and functions (Fig. 1A). For instance, the WW domains have been shown to interact with core components of Hippo pathway, such as LATS1/2 and PTPN14, to facilitate Hippo pathway activation [15–19]. In addition, as a key scaffolding protein enriched in postsynaptic sites, WWC1 can interact with several synaptic proteins including Dendrin and Synaptopodin [20–22]. The PDZ-binding motif and adjacent region of WWC proteins mediate interactions with protein interacting with PRKCA 1 (PICK1), atypical protein kinases (aPKC, PKMζ), and CAMD1, supporting its role in synaptic transmission and memory performance [5, 6, 23, 24]. The C2 domain of WWC1 binds to phosphatidylinositol-3-phosphate (PI3P) enriched on endosome membranes [25]. Moreover, WWC1 has been reported to inhibit the proteasomal degradation of Rab27a, hence playing a role in exosome trafficking and secretion [26]. Unlike WWC1, the roles of WWC2 and WWC3 in the nervous system have been largely unexplored.

Motin family proteins, including AMOT, AMOTL1, and AMOTL2, are also Hippo signaling pathway components (Fig. 1A) [27–31]. *AMOT* gene produces two isoforms, AMOTp130 and AMOTp80, via alternative splicing (Fig. 1A). While AMOT is associated with autism spectrum disorder [32], its role in the nervous system remains poorly understood. In cultured hippocampal neurons, AMOT is enriched in dendritic spines, where it interacts with the actin cytoskeleton and postsynaptic scaffolds, such as multi-PDZ domain protein 1 (MUPP1) and postsynaptic density-95 (PSD-95), and these interactions are critical for actin turnover and stabilization of dendritic spines [33–36]. Moreover, AMOT is indispensable for dendrite growth and arborization in developing neurons, and conditional deletion of *Amot* in mouse neurons results in impaired dendritic network in Purkinje cells and impaired motor coordination [37]. These findings suggest that both WWC proteins and Motins are involved in the regulation of neuronal functions.

The interaction between WWC proteins and Motins has been previously reported [18, 38–42]. In mouse livers deficient in WWC proteins, AMOT protein expression is significantly reduced, although the underlying mechanism has not been studied [38]. Given their significant roles in brain function, it is reasonable to hypothesize that WWC proteins and Motins may coordinate with each other to regulate neuronal functions. Moreover, WWC1 and AMOT can activate Hippo signaling pathway by forming biomolecular condensates in various cellular contexts [43]. In this study, we demonstrate that the protein stability of Motins is regulated by deubiquitinase USP9X in a manner dependent on WWC proteins. Furthermore, we show that the defects in spinogenesis and cognition in mice with neuronal-specific deletion of *Wwc1* and *Wwc2* are partially rescued by ectopic expression of AMOT. Thus, the stabilization of Motins by WWC proteins may participate in the regulation of spinogenesis and cognitive behaviors.

## Results

### WWC1/2/3 regulate protein stability of Motins

To explore whether WWC proteins regulate Motins in different cell lines, we checked the expression of AMOT, AMOTL1, and AMOTL2 in *WWC1/2/3* tKO HEK293A cells. Consistent with the previous report, AMOTp130, and AMOTL1 protein levels were significantly decreased in *WWC1/2/3* tKO cells (Fig. 1B, C) [38]. AMOTp80 was also decreased in some experiments following *Wwc1/2/3* deletion, but this was not consistently observed and might reflect an indirect and context-dependent regulation (Fig. 1B, C). Notably, the expression of AMOTL2 remained largely unchanged (Fig. 1B, C). Similar results were also observed in SH-SY5Y human neuroblastoma cells (Fig. S1A, B). Conversely, AMOTp130 protein level was induced by ectopic expression of WWC1, WWC2, or WWC3 in wild-type or *WWC1/2/3* tKO HEK293A cells (Fig. 1D, S1C). These results indicate that the protein levels of Motins and WWC proteins are positively correlated.

To further investigate how Motins were regulated by WWCs, we examined if the transcription of Motins was perturbed in *WWC1/2/3* tKO cells. As shown in Fig. 1E, the mRNA levels of *AMOT* and *AMOTL1* were not changed upon *WWC1/2/3* deletion, whereas the mRNA level of *AMOTL2* was significantly increased. The increase in *AMOTL2* mRNA level could compensate AMOTL2 protein levels in *WWC1/2/3* tKO cells (Fig. 1B). Since *AMOTL2* is a target gene of YAP/TAZ, this upregulation is likely due to the activation of YAP/TAZ in *WWC1/2/3* tKO cells. Supporting this, we observed an increase in active YAP (aYAP, non-phosphorylated) and TAZ levels, as well as increased expression of canonical YAP/TAZ genes *CTGF*, *CYR61,* and *ANKRD1* (Fig. 1B, E). These results suggest that the downregulation of AMOTp130 and AMOTL1 expression was regulated at a posttranscriptional level.

We then assessed the protein turnover of AMOT in wild-type and *WWC1/2/3* tKO HEK293A cells treated with cycloheximide (CHX, a protein synthesis inhibitor). The half-life of AMOTp130 in wild-type cells was approximately 4 h, whereas it was markedly shortened to 2 h in *WWC1/2/3* tKO cells (Fig. 1F, G). AMOTp80 was much more stable in both wild-type and *WWC1/2/3*-deficient cells (Fig. 1F). These results imply that AMOTp130 is destabilized in the absence of WWC proteins.

### WWC proteins interact directly with and stabilize Motins

**Fig. 2:**
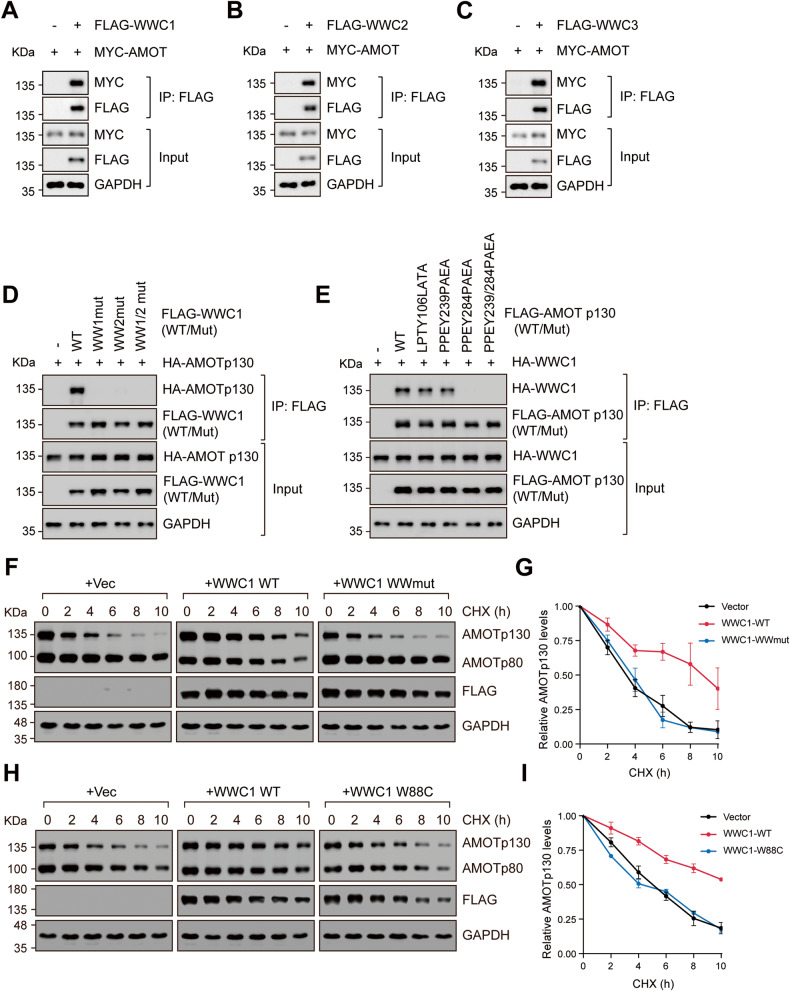
WWC proteins interact directly with and stabilize Motins. **A**–**C** AMOTp130 can interact with WWC1 (**A**), WWC2 (**B**), and WWC3 (**C**). HEK293A cells were co-transfected with the indicated plasmids. Cell lysates were immunoprecipitated (IP) with anti-FLAG beads, and then examined by immunoblotting using the indicated antibodies. **D** Both WW domains in WWC1 mediate interaction with AMOTp130. HA-AMOTp130, WW1mut (W34A/P37A), WW2mut (P84A), WW1/2mut (W34A/P37A/P84A) and WT WWC1 (FLAG-tagged) were expressed in HEK293A cells and subjected to co-immunoprecipitation assays. **E** PPxY motifs in AMOTp130 mediate interaction with WWC1. HA-WWC1, LPTY106LATA, PPEY239PAEA, PPEY284PAEA, PPEY239/284PAEA and WT AMOTp130 (FLAG-tagged) were expressed in HEK293A cells and used for co-immunoprecipitation assay. **F**, **G** WW domains in WWC1 are required for the stabilization of AMOTp130. Whole-cell lysates from vector, WT WWC1, and WWmut WWC1 overexpressed *WWC1/2/3* tKO HEK293A cells treated with cycloheximide (CHX; 100 mg/mL for 2–10 h) were collected and subjected to immunoblotting (**F**). Quantification is shown in **G**. Data are shown as the mean ± SD of three independent experiments. **H**, **I** A patient-derived WWC1 mutant (W88C) is unable to stabilize AMOTp130. WT or W88C mutant WWC1 was overexpressed in *WWC1/2/3* tKO HEK293A cells and treated with cycloheximide (CHX; 100 mg/mL for 2–10 h). Whole-cell lysates were then collected and subjected to immunoblotting (**H**). Quantification is shown in **I**. Data are shown as the mean ± SD of three independent experiments.

Direct interaction between AMOTp130 and WWC1 has been previously reported [18, 38–42]. Indeed, in reciprocal co-immunoprecipitation assays, AMOTp130 interacted with WWC1, WWC2, or WWC3 (Fig. 2A-C and S2A–C). This interaction was abolished when either WW domains in WWC1 were mutated or deleted, or when the third PPxY motif of AMOTp130 was mutated, indicating that the interaction between WWC proteins and Motins was mediated by WW domains in WWC proteins and PPxY motifs in Motins (Fig. 2D, E and S2D, F). On the other hand, the C2 domain and PDZ-binding domain (ADDV) of WWC1/2 were not required for binding with AMOTp130 (Fig. S2D, E). To test whether the interaction between WWC proteins and Motins regulates the protein stability of Motins, the half-life of AMOTp130 in control or WWC1-overexpressing cells was determined. The expression of wild-type WWC1, but not WW domain mutant WWC1, effectively stabilized AMOTp130 (Fig. 2F, G). The tryptophan residue in WWC1 at position 88 (W88) is critical for the hydrophobic interaction between WW domains and PPxY motifs. The W88C mutation in patients with Tourette syndrome has been reported to affect the formation of the WWC1-Dendrin complex [20]. We found that the interaction of WWC1 W88C mutant with AMOTp130 was reduced compared with wild-type WWC1 (Fig. S2F, G). Consistently, the WWC1 W88C mutant did not significantly boost the stability of AMOTp130 (Fig. 2H, I). Together, these results indicate that WWC proteins interact directly with Motins, and this interaction is crucial for regulating the protein stability of Motins.

### WWC proteins recruit USP9X to deubiquitinate and stabilize Motins

**Fig. 3:**
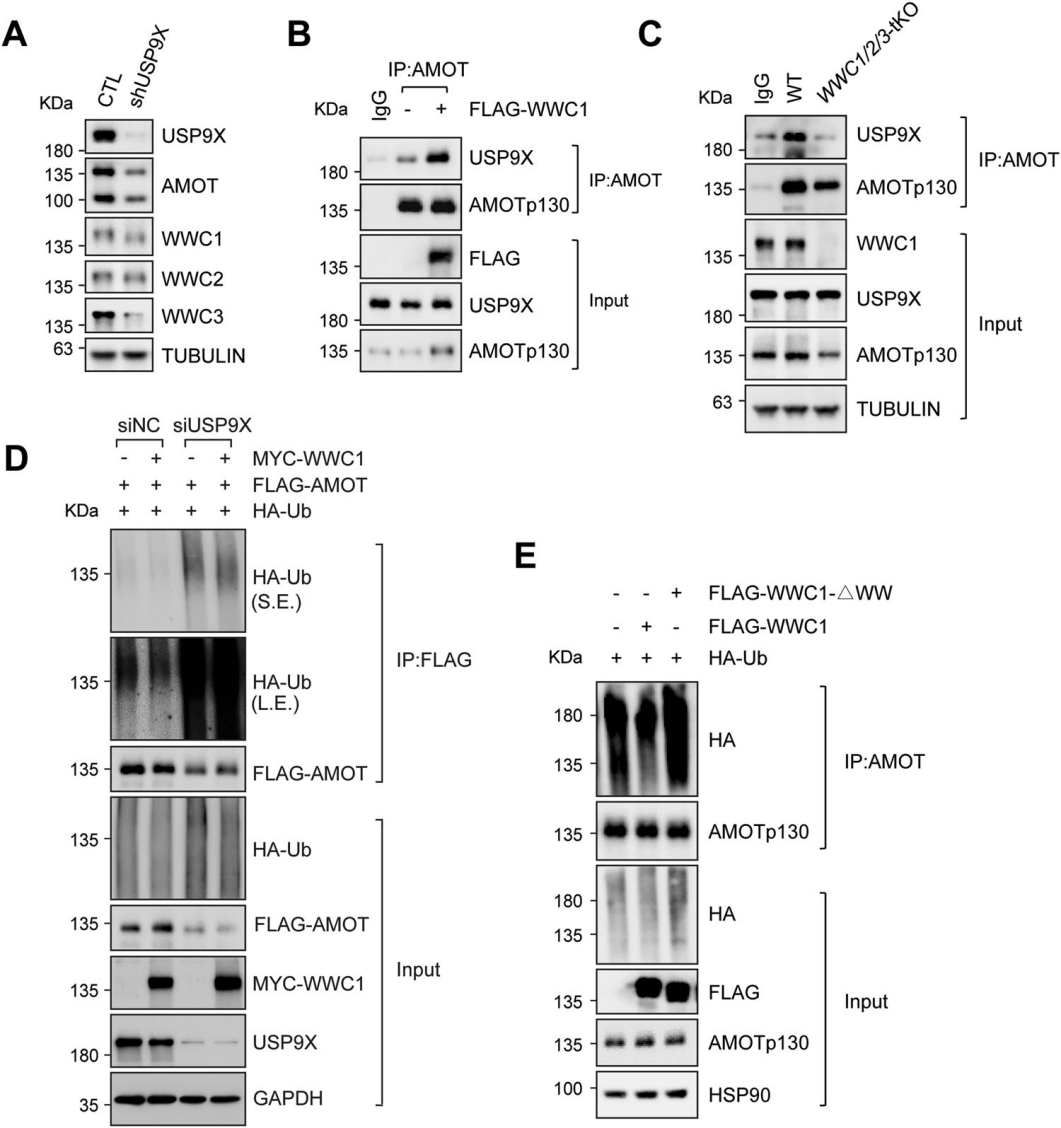
WWC proteins recruit USP9X to deubiquitinate and stabilize Motins. **A** AMOT protein level is reduced in *USP9X* knockdown HEK293A cells. Knockdown was achieved by expression of shRNA targeting *USP9X*. **B** The interaction between AMOT and USP9X is enhanced by ectopic WWC1 expression. Endogenous AMOT was immunoprecipitated from HEK293A cells with or without WWC1 overexpression. Precipitated proteins were examined by immunoblotting. **C** The interaction between AMOT and USP9X is repressed in *WWC1/2/3*-deficient cells. Endogenous AMOT was immunoprecipitated from wild-type or *WWC1/2/3* tKO HEK293A cells and precipitated proteins were examined by immunoblotting. **D** WWC1 is required for AMOT deubiquitination by USP9X. HEK293A cells were co-transfected with indicated siRNA, FLAG-AMOTp130, HA-ubiquitin (Ub), and/or MYC-WWC1. Cell lysates were subjected to immunoprecipitation and immunoblotting. **E** WW domains of WWC1 are required for AMOT deubiquitination. Cell lysates from HEK293A cells co-transfected with HA-Ub and WWC1 (wild-type) or WWC1 mutant (WWC1-△WW) (FLAG-tagged) were treated with 10 μM MG132 for 4 h and subjected to immunoprecipitation and immunoblotting.

The ubiquitin-proteasome system (UPS) is responsible for regulating cellular protein turnover and homeostasis [44, 45]. E3 ligases such as RNF146 and NEDD4 have been shown to promote ubiquitination and proteasomal degradation of AMOT [29, 46, 47]. Conversely, USP9X deubiquitinase stabilizes multiple Hippo pathway components, including LATS2, WWC1, and AMOTp130 [48–51]. Indeed, the protein levels of AMOTp130 and WWC proteins were decreased in *USP9X* knockdown HEK293 A cells (Fig. 3A). Since WWC proteins regulate the protein stability of Motins, and both are targeted by USP9X, we propose that USP9X may play a role in the stabilization of Motins by WWC proteins.

As indicated by co-immunoprecipitation assays, USP9X interacted with both AMOTp130 and WWC1 (Fig. 3B, S3A). Interestingly, in the presence of ectopic WWC1, the interaction between AMOTp130 and USP9X was significantly strengthened (Fig. 3B). Conversely, the interaction between AMOTp130 and USP9X was decreased in *WWC1/2/3* tKO cells (Fig. 3C). Moreover, the ubiquitination of AMOTp130 was significantly induced in *USP9X*-knockdown cells, and ectopic expression of WWC1 was able to reduce the ubiquitination of AMOTp130 in wild-type but not *USP9X*-knockdown cells (Fig. 3D). Furthermore, we observed that the expression of wild-type WWC1, but not WW domain mutant WWC1, effectively reduced the ubiquitination of AMOTp130 (Fig. 3E). Consistently, the half-life of AMOTp130 was shortened in *USP9X*-deficient cells (Fig. S3B, C). These results indicate that WWC proteins, as a bridge between AMOTp130 and USP9X, reduce ubiquitination and then stabilize AMOTp130.

### WWC proteins regulate stability of Motins in neurons and brain

**Fig. 4:**
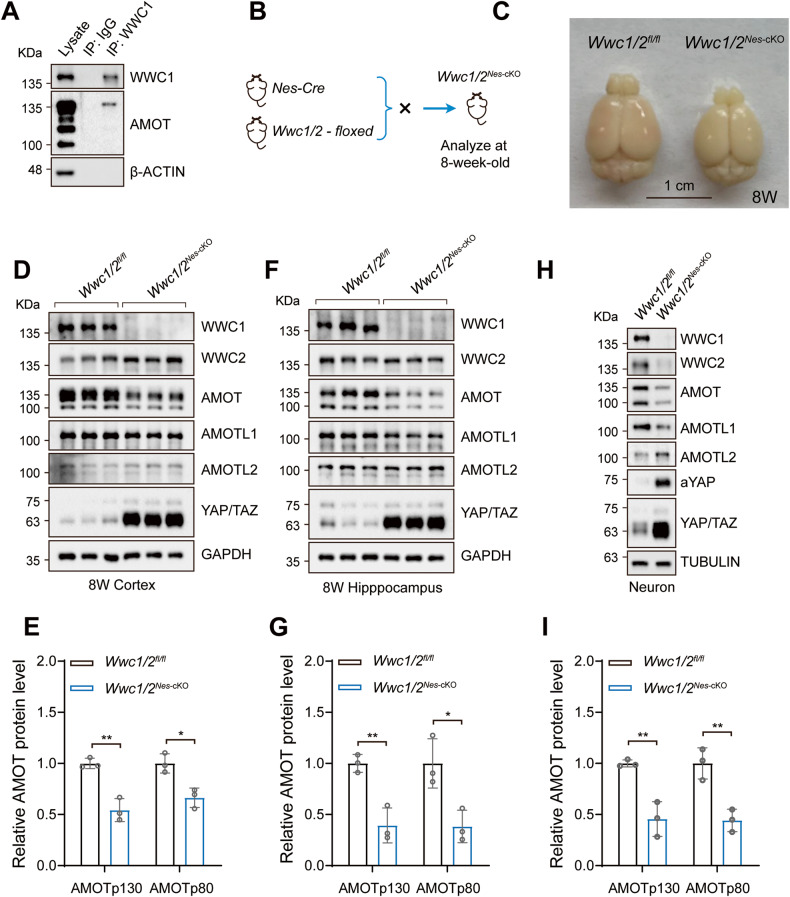
WWC proteins regulate the stability of Motins in neurons and brain. **A** AMOT is coprecipitated with WWC1 from mouse cortex homogenates extracted from wild-type mouse brain at P21. IgG was used as the control. **B** *Wwc1/2*^*Nes*-cKO^ mouse model. *Wwc1/2*^*fl/fl*^ mice were crossed with *Nes*-Cre mice to generate *Wwc1/2*^*Nes*-cKO^ mice. **C** Representative whole-mount image of 8-week-old *Wwc1/2*^*fl/fl*^ and *Wwc1/2*^*Nes*-cKO^ mouse brains is shown. **D**, **E** Downregulation of AMOTp130 in *Wwc1/2*^*Nes*-cKO^ cortex. Immunoblots (**D**) and quantification (**E**) of indicated proteins in cortical tissues from *Wwc1/2*^*fl/fl*^ and *Wwc1/2*^*Nes*-cKO^ mice (*n* = 3 per genotype) at 8 W are shown. A short WWC2 might be alternatively translated after DNA recombination, which lacks WW domains and should be inactive. Data are shown as the mean ± SD. *p < 0.05, **p < 0.01 between indicated groups. **F**, **G** Downregulation of AMOTp130 in *Wwc1/2*^*Nes*-cKO^ hippocampus. Immunoblots (**F**) and quantification (**G**) of indicated proteins in hippocampal tissues from *Wwc1/2*^*fl/fl*^ and *Wwc1/2*^*Nes*-cKO^ mice (*n* = 3 per genotype) at 8 W are shown. Data are shown as the mean ± SD. *p < 0.05, **p < 0.01 between indicated groups. **H**, **I** Levels of Motins are decreased in *Wwc1****/****2* dKO primary cortical neurons isolated from *Wwc1/2*^*fl/fl*^ and *Wwc1/2*^*Nes*-cKO^ mice (**H**). Quantification is shown in **I**. Data are shown as the mean ± SD of three independent experiments; **p < 0.01 between indicated groups.

Both WWC1 and AMOT are involved in regulating neuronal functions [4–7, 9, 11, 12, 20, 33, 37, 52]. We then asked whether the functions of WWC1 and AMOT in the nervous system were coupled. WWC1 is widely expressed in multiple brain regions, particularly in the hippocampus and cortex [53]. Nevertheless, it is unclear whether WWC2, USP9X, and Motins share similar expressions and functions in the nervous system, although abundant mRNA levels of these genes are detected in brain cells (Allen brain atlas) [37, 54, 55]. Western blot analysis revealed that WWC1/2 (no *Wwc3* gene in mice), USP9X, and AMOT were ubiquitously expressed in the mouse brain, with relatively higher expression in cortex and hippocampus, regions highly related to memory function (Fig. S4A). These proteins were also highly expressed in primary cultured cortical neurons, and their expression gradually increased during neuronal maturation (Fig. S4B). Neuronal maturation is accompanied by spinogenesis, the process of dendritic spine development in neurons [56]. Supporting the role of WWC proteins and AMOT in spinogenesis, WWC proteins, and AMOT were mainly enriched in postsynaptic density (PSD) fractions following sucrose gradient fractionation of mouse cortex or hippocampus (Fig. S4C, D), which was consistent with previous reports [5, 33, 53, 57]. In mouse cortex extracts, WWC1 also interacted with AMOTp130 at the endogenous level, suggesting a conserved regulatory mechanism in the mouse brain (Fig. 4A).

To explore the role of WWC proteins in the stability of Motins in the mammalian brain, we first generated brain-specific *Wwc1/2* knockout (KO) mice (*Wwc1/2*^*Nes*-cKO^) by crossing *Wwc1/2* flox mice with *Nestin*-Cre mice, the latter drove DNA recombination specifically in apical neural progenitor cells (NPCs) starting on E10.5 (Fig. 4B) [15, 58]. *Wwc1/2*^*Nes*-cKO^ mice were born normal but exhibited growth retardation, as indicated by smaller body size and brain size (Fig. 4C and S4E, F). We dissected the cortex and hippocampus of 8-week-old *Wwc1/2*^*Nes*-cKO^ and control mice, and observed efficient inactivation of WWC1/2 (a short WWC2 might be alternatively translated after DNA recombination, which lacks WW domains and should be inactive). Interestingly, in *Wwc1/2*^*Nes*-cKO^ mice, the protein level of AMOTp130, and to a less extent AMOTp80 and AMOTL1, was reduced, albeit no significant change at the mRNA level was observed (Fig. 4D–G and S4G, H). The expression of TAZ was also significantly induced in *Wwc1/2*^*Nes*-cKO^ mice, which may contribute to abnormal brain development and was not further investigated in this study (Fig. 4D, F). We isolated the primary neurons from *Wwc1/2*^*Nes*-cKO^ and control mice and observed a reduction in AMOT and AMOTL1 expression (Fig. 4H, I). Moreover, expression of wild-type WWC1, but not WW domain mutant and W88C WWC1, rescued AMOTp130 expression in *Wwc1/2*^*Nes*-cKO^ primary neurons (Fig. S4I, J). Collectively, these results indicate that the protein stability of Motins, especially AMOTp130, and AMOTL1, is regulated by WWC proteins in the brain and neurons.

### Defective spinogenesis and cognition in WWC1/2-deficient mice

**Fig. 5:**
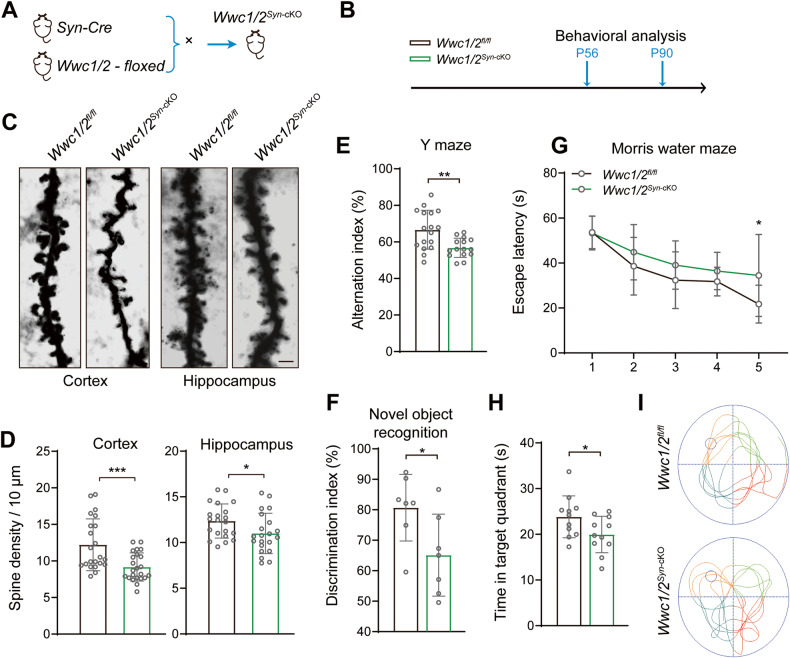
Defective spinogenesis and cognition in WWC1/2-deficient mice. **A**, **B** Strategy for *Wwc1/2*^*Syn*-cKO^ mouse model and subsequent behavioral tests. *Wwc1/2*^*fl/fl*^ mice were crossed with *Syn*-Cre mice to generate *Wwc1/2*^*Syn*-cKO^ mice (**A**). Timeline of the indicated behavioral tests in *Wwc1/2*^*fl/fl*^ and *Wwc1/2*^*Syn*-cKO^ mice (**B**). **C** Representative images of dendritic branches of Golgi-stained prefrontal cortex pyramidal neurons (left) and hippocampal neurons (right) from *Wwc1/2*^*fl/fl*^ and *Wwc1/2*^*Syn*-cKO^ mice are shown. Scale bar, 2 μm. **D** Quantifications of dendritic spine density of the pyramidal neurons in the prefrontal cortex region (left, *Wwc1/2*^*fl/fl*^, *n* = 22; *Wwc1/2*^*Syn*-cKO^, *n* = 23) and hippocampal neurons in the dentate gyrus region (right, *Wwc1/2*^*fl/fl*^, *n* = 21; *Wwc1/2*^*Syn*-cKO^, *n* = 21) are shown. Data are shown as the mean ± SD; *p < 0.05, ***p < 0.001 between indicated groups. **E** *Wwc1/2* deficiency is associated with impaired sequential alternations in Y maze test. *Wwc1/2*^*fl/fl*^, *n* = 17; *Wwc1/2*^*Syn*-cKO^, *n* = 15. Data are shown as the mean ± SD; **p < 0.01 between indicated groups. **F** *Wwc1/2* deficiency is associated with impaired discrimination index in the novel object recognition test. *Wwc1/2*^*fl/fl*^, *n* = 7; *Wwc1/2*^*Syn*-cKO^, *n* = 7. Data are shown as the mean ± SD; *p < 0.05 between indicated groups. **G**–**I** *Wwc1/2* deficiency is associated with impaired performance in the Morris water maze test. Quantifications of average time spent to reach the hidden platform during the 5 days training session (**G**), average time spent in target quadrant when the platform was absent (**H**), and representative path traces at day 5 (**I**) are shown. *Wwc1/2*^*fl/fl*^, *n* = 11; *Wwc1/2*^*Syn*-cKO^, *n* = 11. Data are shown as the mean ± SD; *p < 0.05 between indicated groups.

The growth defects of *Wwc1/2*^*Nes*-cKO^ mice prevented in-depth analysis of neuronal functions associated with *Wwc1/2* deletion. To specifically delete *Wwc1/2* in neurons, we crossed *Wwc1/2* flox mice with transgenic mice expressing Cre recombinase under Synapsin 1 regulatory element (*Syn*-Cre mice) (Fig. 5A, B). *Wwc1/2*^*Syn*-Cre^ mice exhibited normal growth and brain size, and H&E staining on brain sections revealed no significant morphological differences including cortex thickness (Fig. S5A–D). Moreover, immunofluorescence staining of superficial and deep neocortical layers indicated that the thickness of the cortex and neuronal densities of *Wwc1/2*^*Syn*-cKO^ mice was comparable to that of control littermates (Fig. S5E), indicating that cortical development and neurogenesis were largely unaffected by *Wwc1/2* deletion in neurons. We also analyzed the spine densities of neurons in the prefrontal cortex and hippocampal dentate gyrus in *Wwc1/2*^*Syn*-cKO^ mice using Golgi staining. Compared to the control mice, the dendritic spine density was reduced in *Wwc1/2*^*Syn*-cKO^ mice (Fig. 5C, D). These results suggest that mice with *Wwc1/2* deletion in neurons have normal brain morphology, but the brain function is affected due to defective spinogenesis.

WWC1 (KIBRA) plays a role in synaptic plasticity, learning, and memory [4, 6, 20]. To determine the effect of *Wwc1/2* deficiency on brain function, we performed multiple behavioral tests on *Wwc1/2*^*Syn*-cKO^ mice and control littermates (Fig. 5B). In the Y maze test, *Wwc1/2*^*Syn*-cKO^ mice exhibited reduced sequential alternations, indicating impaired associative working memory (Fig. 5E). Consistently, in the novel object recognition test, *Wwc1/2*^*Syn*-cKO^ mice showed no preference for the novel objects, indicating a defect in recognition memory (Fig. 5F). Previously, it has been shown that *Wwc1* KO mice displayed extensive deficits in spatial learning and memory [6, 20]. We asked whether similar defects existed in *Wwc1/2*^*Syn*-cKO^ mice. In the Morris water maze test, *Wwc1/2*^*Syn*-cKO^ mice exhibited increased latency to reach a hidden platform, reduced time spent in the platform quadrant, and longer swim-path traces to reach the hidden platform on day 5 (Fig. 5G–I), suggesting impairment in spatial learning and memory. Together, these data support the role of WWC1/2 in regulating cognitive function in mice.

We also performed additional tests to monitor behaviors like depression and locomotor coordination, but no significant difference was observed between *Wwc1/2*^*Syn*-cKO^ mice and control mice. For instance, *Wwc1/2*^*Syn*-cKO^ and control mice showed comparable moving velocity and spent similar time at the center in the open field test, although *Wwc1/2*^*Syn*-cKO^ mice traveled a slightly longer distance (Fig. S5F-I). In the elevated plus maze test, *Wwc1/2*^*Syn*-cKO^ mice showed similar exploring time in the open arms, but relatively less time in the closed arms, compared with control mice (Fig. S5J, K). Moreover, *Wwc1/2*^*Syn*-cKO^ and control mice showed similar immobility time in the tail suspension test and latency to fall in the rotarod test (Fig. S5L, M). Hence, the poor performance in learning and memory-related assays observed in *Wwc1/2*^*Syn*-cKO^ mice was not due to a deficiency in motor ability. Taken together, these data suggest that *Wwc1/2* deletion has no significant impact on neuropsychiatry-associated functions and motor coordination.

### The phenotype of WWC1/2 loss is partially rescued by ectopic expression of AMOT

**Fig. 6:**
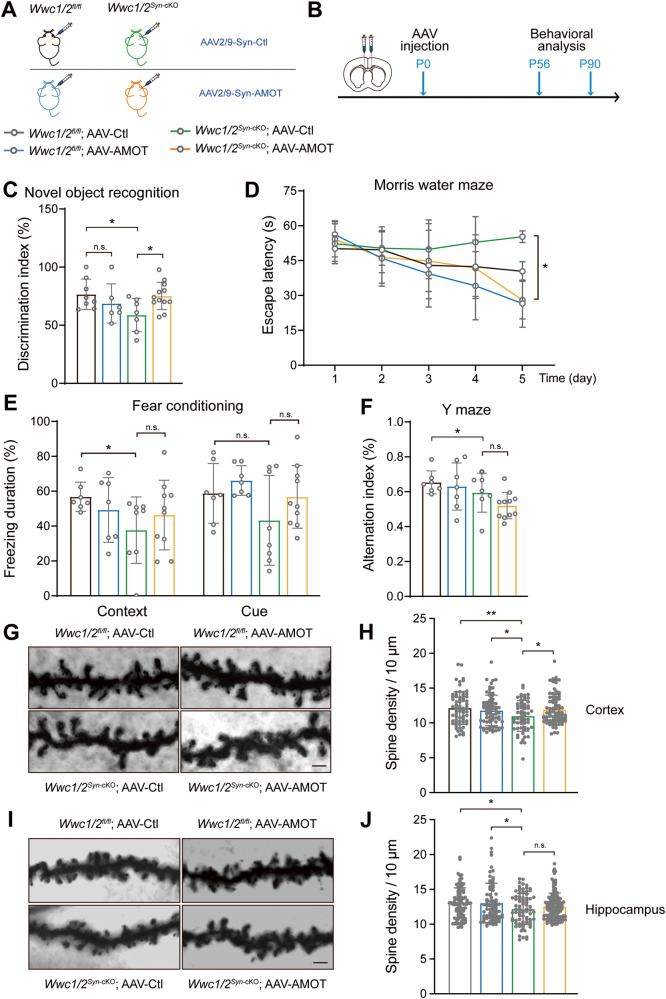
The phenotype of WWC1/2 loss is partially rescued by ectopic expression of AMOT. **A** Schematic illustration of experiment design. *Wwc1/2*^*fl/fl*^ mice injected with AAV2/9-vector (*Wwc1/2*^*fl/fl*^; AAV-Ctl) or AAV2/9-AMOT (*Wwc1/2*^*fl/fl*^; AAV-AMOT) and *Wwc1/2*^*Syn*-cKO^ mice injected with AAV2/9-vector (*Wwc1/2*^*Syn*-cKO^; AAV-Ctl) or AAV2/9-AMOT (*Wwc1/2*^*Syn*-cKO^; AAV-AMOT) were indicated by different colors. The color scheme was used throughout this figure. **B** Timeline of the adeno-associated virus (AAV) injection and subsequent behavioral tests in *Wwc1/2*^*fl/fl*^ and *Wwc1/2*^*Syn*-cKO^ mice injected with the indicated viruses. **C** Ectopic expression of AMOT ameliorates the performance of *Wwc1/2*^*Syn*-cKO^ mice in novel object recognition test. The discrimination index was quantified. *Wwc1/2*^*fl/fl*^ mice were injected with AAV2/9-vector (*n* = 8) or AAV2/9-AMOT (*n* = 6); *Wwc1/2*^*Syn*-cKO^ mice were injected with AAV2/9-vector (*n* = 7) or AAV2/9-AMOT (*n* = 12). Data are shown as the mean ± SD; *p < 0.05, n.s. (not significant) between indicated groups. **D** Ectopic expression of AMOT ameliorates the performance of *Wwc1/2*^*Syn*-cKO^ mice on day 5 in the Morris water maze test. Quantification of average time spent to reach the hidden platform during the 5 days training session is shown. *Wwc1/2*^*fl/fl*^ mice were injected with AAV2/9-vector (*n* = 9) or AAV2/9-AMOT (*n* = 7); *Wwc1/2*^*Syn*-cKO^ mice were injected with AAV2/9-vector (*n* = 9) or AAV2/9-AMOT (*n* = 12). Data are shown as the mean ± SD; *p < 0.05 between indicated groups. **E** Fear conditioning test. Contextual fear-conditioned memory was assessed by time spent in freezing during a 6-minute exposure to the same context and cued fear-conditioned memory was assessed by time spent in freezing during a 3-minute exposure to the tone presented in a novel context. *Wwc1/2*^*fl/fl*^ mice were injected with AAV2/9-vector (*n* = 7) or AAV2/9-AMOT (*n* = 7); *Wwc1/2*^*Syn*-cKO^ mice were injected with AAV2/9-vector (*n* = 8) or AAV2/9-AMOT (*n* = 10). Data are shown as the mean ± SD; *p < 0.05, n.s. (not significant) between indicated groups. **F** Y maze test. The percentage of sequential alternations was quantified. *Wwc1/2*^*fl/fl*^ mice were injected with AAV2/9-vector (*n* = 7) or AAV2/9-AMOT (*n* = 7); *Wwc1/2*^*Syn*-cKO^ mice were injected with AAV2/9-vector (*n* = 7) or AAV2/9-AMOT (*n* = 11). Data are shown as the mean ± SD; *p < 0.05, n.s. (not significant) between indicated groups. **G**, **I** Representative images of dendritic branches of Golgi-stained prefrontal cortex pyramidal neurons (**G**) and hippocampal dentate granule neurons (**I**) from four groups of mice (*n* = 4 per group). Scale bar, 2 μm. **H**, **J** Quantifications of dendritic spine density of the pyramidal neurons in the prefrontal cortex region (**H**) and the granular neurons in the hippocampal dentate gyrus region (**J**) are shown. Each data point indicates spine density of a dendrite counted. ((**H**), left to right, *n* = 70, 71, 59, 78; (**J**), left to right, *n* = 93, 75, 79, 128). Data are shown as the mean ± SD; *p < 0.05, **p < 0.01, and n.s. (not significant) between indicated groups.

The reduced expression of AMOT in *Wwc1/2* KO neurons may contribute to the phenotypes associated with *Wwc1/2* deficiency (Fig. 4H). We tested whether ectopic expression of AMOT could rescue the impaired neuronal functions in *Wwc1/2* KO mice. We injected AAV2/9-hSyn-ZsGreen viruses—which expressed ZsGreen under the control of a Synapsin promoter—into the lateral ventricles of neonatal (P0) pups. This led to a widespread and robust neuronal expression of ZsGreen in the cortex and hippocampus, and weak expression in cerebellar granule neurons (Fig. S6A). AAV2/9 viruses expressing either AMOTp130 (AAV2/9-AMOT) or control fluorescent protein ZsGreen (AAV2/9-Ctl) were then delivered to the lateral ventricles of *Wwc1/2*^*Syn*-cKO^ or control mice. Viral injection had no significant effect on the growth and brain development of mice (Fig. S6B, C). Immunoblotting analysis confirmed the expression of FLAG-tagged AMOTp130 in the cortex and hippocampus of most mice injected with AAV2/9-AMOT (Fig. S6D–G). Subsequently, we carried out a range of behavioral tests to determine the role of AMOTp130 in cognitive functions related to WWC1/2 (Fig. 6A, B). In the novel object recognition test, AAV2/9-AMOT injection could effectively normalize the impaired recognition memory in *Wwc1/2*^*Syn*-cKO^ mice (Fig. 6C). AAV2/9-AMOT injection in *Wwc1/2*^*Syn*-cKO^ mice also remarkably shortened the time spent in latency to find the hidden platform in the Morris water maze, suggesting that spatial learning and memory could be partially rescued by overexpression of AMOT (Fig. 6D). Moreover, AAV2/9-AMOT injection in *Wwc1/2*^*Syn*-cKO^ mice exhibited a tendency of improved performance under context condition, although the change was not statistically significant (Fig. 6E). On the other hand, AAV2/9-AMOT injection failed to restore working memory and associative memory, as indicated by the Y maze test (Fig. 6F). To assess the role of AMOT in WWC1/2-mediated spinogenesis, we also analyzed the spine density of neurons in the prefrontal cortex and hippocampal dentate gyrus from four groups of mice (Fig. 6G–J). In cortical neurons, AMOT overexpression significantly improved defects in spinogenesis (Fig. 6G, H). While the spine density of *Wwc1/2*^*Syn*-cKO^ mice was reduced in hippocampal neurons, there was also a tendency of recovery in mice injected with AAV2/9-AMOT (Fig. 6I, J). WWC1 is well known to regulate the activity and expression of α-amino-3-hydroxyl-5-methyl-4-isoxazole-propionate receptors (AMPA receptors), which are crucial for learning and memory [4, 5, 24, 59, 60]. Our results indicated that overexpression of AMOT in *Wwc1/2* KO neurons induced GluA1 and GluA2 expression (Fig. S6H, I), indicating a potential role of AMOT in AMPAR regulation. Together, the expression of AMOTp130 could ameliorate impaired cognitive functions associated with WWC1/2 deficiency. However, likely due to the heterogeneous spatiotemporal expression of AMOTp130 following AAV2/9 injection, complete rescue of neuronal phenotypes caused by *Wwc1/2* deletion was not achieved.

## Discussion

In this study, we reveal a molecular mechanism underlying the stabilization of Motins by WWC proteins. WWC proteins positively regulate Motins, including AMOTp130 and AMOTL1, which is consistent with a previous study [38]. AMOTL2 might be regulated both post-translationally by WWC proteins and transcriptionally by YAP/TAZ. In addition, we have shown that WWC proteins bind directly to Motins through an interaction mediated by WW domains and PPxY motifs on WWC proteins and Motins, respectively. Moreover, WWC proteins are required for Motins to recruit protein deubiquitinase USP9X, remove polyubiquitination, and prevent protein degradation. USP9X also regulates the protein stability of WWC proteins [48] (Fig. 3). Thus, the cellular levels of WWC proteins and Motins are positively correlated, suggesting that these two proteins may jointly regulate common biological processes.

Dendritic spinogenesis and pruning are fundamental to synapse connections, neural circuit wiring, and subsequent higher brain function. In neurons, the protein stability of Motins is also tightly coupled with WWC proteins. Both Motins and WWC proteins are highly enriched in the dendritic spine, specifically in postsynaptic density (Fig. S4). Indeed, *Wwc1/2* deletion in neurons leads to a sparse dendritic spine, which likely contributes to aberrant neurobehavioral features observed in *Wwc1/2*^*Syn*-cKO^ mice, in particular, impaired learning and memory. (Fig. 5). Notably, the defects caused by *Wwc1/2* deficiency are partially rescued by ectopic expression of AMOTp130 (Fig. 6). Hence, Motins and WWC proteins coordinate the regulation of dendritic spine and cognitive functions.

The remodeling of actin-microtubule cytoskeleton is pivotal in regulating dendritic spine plasticity [61]. For instance, the nucleation, elongation, and branching of actin filaments are involved in the maturation and stabilization of the dendritic spine [62, 63]. Moreover, microtubules are present in dendritic spine and regulate their morphology and synaptic plasticity [64, 65]. Interestingly, Motins interact with actin filaments [27, 28, 66–68], and WWC1 has been shown to associate with the microtubule motor protein dynein light chain 1 [69], which is involved in endocytic recycling compartment transport by the SNX4-WWC1-dynein complex [70]. Hence, Motins and WWC proteins together may serve as an actin-microtubule adaptor to coordinate the dynamics of dendritic spine and synaptic plasticity [71]. In addition to Motins, WWC proteins also interact with additional components of the dendritic spine, such as dendrin, PICK1, synaptopodin, and aPKC isoforms [5, 6, 20, 22, 23, 72]. It is also possible that WWC proteins are involved in the transport of multiple regulators of the dendritic spine to defined subcellular locations. Moreover, a recent study has suggested that AMOT and WWC1 form condensates in response to distinct signals, it would be compelling to investigate whether such condensates are involved in spinogenesis [43].

Both WWC proteins and Motins are important Hippo pathway regulators, and their downregulation leads to activation of downstream effectors YAP/TAZ [1, 2, 15, 38, 48, 51, 73–79]. Our study found that YAP/TAZ target genes were significantly upregulated in *Wwc1/2* KO neurons. However, *Amot* deletion in neurons did not significantly affect YAP target genes expression [37], suggesting a Hippo pathway-independent role of AMOT. Moreover, a WWC1 mutant defective in LATS binding was found to increase WWC1 abundance in AMPAR complexes, improving hippocampal-dependent learning and memory [59]. This suggests a potential link between the Hippo pathway, WWC1, and memory performance. It is currently unclear whether the coupling of the protein levels of WWC proteins and Motins regulates YAP/TAZ activity, and whether YAP/TAZ activation in *Wwc1/2* KO mice accounts for the impaired dendritic spine and cognitive functions. A side-by-side comparison of YAP/TAZ loss-of-function and AMOT gain-of-function on the *Wwc1/2* KO background should clarify these questions.

## Materials and methods

### DNA constructs, lentivirus production, and infection

The full-length open reading frame (ORF) and truncations with FLAG/HA/MYC tag were amplified by polymerase chain reaction (PCR) and cloned into pLVX vector (632164, Takara, Japan) using ClonExpress MultiS One Step Cloning Kit (C113–02, Vazyme, Nanjing, China). FLAG-tagged full-length Amot was constructed by cloning the corresponding mouse cDNA into pLVX vector. For CRISPR/Cas9 cloning, sgRNA oligos were cloned into the lentiCRISPR v2 vector provided by Dr. Feng Zhang [80], using T4 ligase (2011A, Takara, Shiga, Japan). PLVX or lentiCRISPR coupled with packaging vectors psPAX2 and pMD2.g were co-transfected into HEK293T cells to produce a high titer lentivirus. After transfection for 48 h, medium supernatant was harvested and filtered through a 0.45 μm filter (SLHP033RS, Millipore, MD, USA). To establish stable cell lines, cells were infected with the lentivirus concentrated by PEG8000 in the presence of 10 μg/ml polybrene (TR-1003-G, Sigma-Aldrich, MO, USA) and cells were selected with 2 μg/ml puromycin (ant-pr-1, InvivoGen, Toulouse, France) 48 h after infection.

### Cell lines and DNA transfection

HEK293A cells were maintained in DMEM (GIBCO, Waltham, ME, USA) and SH-SY5Y cells in DMEM/F12 (GIBCO) media. Media were supplemented with 10% (vol/vol) fetal bovine serum (FBS, GIBCO) and 50 mg/ml P/S (penicillin/streptomycin, SV30010, Hyclone, Logan, UT, USA). All cell lines were authenticated according to the short tandem repeat (STR) profile. The sgRNA sequences targeting individual genes are listed in Supplementary Table 1. *WWC1/2/*3 tKO cells were described previously [15]. Cell lines were transfected with indicated plasmids using PolyJet Transfection Reagent (SL100688, SignaGen Laboratories, Rockville, MD, USA) according to the manufacturer’s instructions.

### siRNA transfection

siRNA for USP9X and non-targeting control were synthesized by HANBIO (Shanghai, China) and prepared at a concentration of 20 μM in nuclease-free water. siRNA transfection was conducted using Lipofectamine RNAiMAX Transfection reagent (Cat. No. 13778075, Invitrogen, NY, Empire State, USA) according to the manufacturer’s instructions. The siRNA sequences are listed in Supplementary Table 1.

### Primary cortical neuronal culture

Male and female C57BL/6 mice were used in this assay. Cortical tissues of E14.5-E15.5 *Wwc1/2*^*fl/fl*^/*Wwc1/2*^*Nes*-cKO^ mice were dissected and then digested with papain (Cat. No. LS003126, Worthington Bio Corp, Lakewood, NJ, USA) for 30 min at 37 °C to generate dissociated neurons for cell culture. Cells were seeded at a density of 1 × 10^5^ per square centimeter onto 6-well plates pre-coated with poly-L-lysine containing Neurobasal medium (Cat. No. 21103049, Invitrogen, USA) supplemented with B-27 supplement (Cat. No. 17504044, Gibco, USA), 2 mM Glutamax (Cat. No. 35050061, Invitrogen, USA) and 1% penicillin/streptomycin (SV30010, Hyclone, USA). Primary neurons were then maintained at 37 °C in a humidified atmosphere (95% air and 5% CO2), and half of the medium was refreshed every two days.

### RNA extraction and real-time qPCR

Total RNA was isolated from cells or tissue samples using the MiniBEST Universal RNA Extraction Kit (TaKaRa, Japan), and cDNA was synthesized using the First-Strand cDNA Synthesis SuperMix (TransGen Biotech, Beijing, China). Quantitative real-time PCR was conducted by using TB Green® *Premix Ex Taq* (TaKaRa, Japan) on a CFX96 Real-Time PCR system (Bio-Rad, USA). Primer sequences used in this study are listed in Supplementary Table 1. Relative mRNA levels were normalized to the housekeeping gene *β-ACTIN*. All reactions were performed in biological triplicates.

### Immunoblotting

Cultured cells and brain tissues were lysed and homogenized in radioimmunoprecipitation assay (RIPA) buffer (50 mM HEPES (pH7.5), 150 mM NaCl, 1% TritonX-100, 0.1% SDS, 0.5% Sodium Deoxycholate, 1 mM PMSF, protease inhibitor cocktail (HY-K0010, MCE, NJ, USA) and phosphatase inhibitors (HY-K0021, MCE, USA)). Postsynaptic density fractions from adult brain tissues were isolated as previously described [4, 53]. Proteins were separated by sodium dodecyl sulfate-polyacrylamide gel electrophoresis (SDS-PAGE) and transferred to nitrocellulose membranes. After blocking with 5% non-fat milk in Tris-buffered saline with 0.1% Tween, the membranes were incubated with indicated primary antibodies in 5% bovine serum albumin (BSA) overnight at 4 °C and then appropriate horseradish peroxidase-conjugated secondary antibodies in 5% non-fat milk for 1 h at room temperature. Bands were visualized using High-sig ECL Western Blotting Substrate (#180–501, Tanon, Shanghai, China) and Tanon 5200 S imaging system. The intensities of protein bands were quantified using ImageJ software (NIH).

### Immunoprecipitation

Cultured cell lysates extracted from mild lysis buffer (50 mM HEPES at pH7.5, 150 mM NaCl, 1 mM EDTA, 1% NP-40, 50 mM NaF, 0.5% Sodium Deoxycholate, 1 mM PMSF, protease inhibitor cocktail (HY-K0010, MCE, USA) and phosphatase inhibitors (HY-K0021, MCE, USA)) were centrifuged at 12,000 rpm for 15 min at 4 °C, and then the supernatants were incubated with Anti-DYKDDDDK Affinity Beads (SA042001, Smart Lifesciences, Changzhou, China) with rotation overnight at 4 °C. After washing with ice-cold mild lysis buffer 4 times, the complexes were resuspended in SDS loading buffer (50 mM Tris-HCl at pH6.8, 2% SDS, 10% glycerol, 0.025% bromophenol blue and β-mercaptoethanol) at 95 °C for 5 min, followed by immunoblotting for analysis.

Mouse brain tissues were homogenized and lysed with RIPA buffer. The lysates were then rotated at 4 °C for 30 min, and centrifuged for 20 min at 12,000 rpm at 4 °C. The supernatants were incubated with the primary antibody for 1 h at 4 °C. Then protein A/G agarose beads (sc-2003, Santa Cruz, CA, USA) were added to the lysates and incubated with rotation at 4 °C for 2 h. After washing with ice-cold RIPA buffer, the complexes were resuspended in SDS loading buffer for 5 min at 95 °C, and the supernatant was subsequently subjected to SDS-PAGE for immunoblotting analysis.

### Protein degradation and ubiquitination assay

To detect the protein degradation of AMOT, cells were treated with cycloheximide (CHX, 100 μg/ml, MCE, USA) at different time points before harvesting for immunoblotting analysis. To detect the ubiquitination level of AMOTp130, cells were transfected with pCDNA-HA-Ub, and harvested 48 h after transfection. Before harvest, cells were treated with MG132 (10 μM) for 4 h. Protein lysates were extracted by SDS lysis buffer (50 mM Tris-HCl at pH7.5; 1% SDS and 10 mM DTT) and boiled at 95 °C for 10 min. The lysates were diluted 10 times with mild lysis buffer and subjected to immunoprecipitation and immunoblotting.

### Immunohistochemistry

For immunofluorescence staining, age-matched mice were perfused with phosphate-buffered saline (PBS) followed by 4% paraformaldehyde (wt/vol) in PBS. Brains were removed from the skull and kept in 4% paraformaldehyde (wt/vol) in PBS. After fixation, the brain was equilibrated in 30% sucrose at 4 °C, embedded in Tissue-Tek O.C.T. Compound (SAKURA, Japan), and coronally sectioned at 25 μm with a cryostat (Leica 1950 Ag Protect, Leica, Wetzlar, Germany). After incubation in blocking buffer (10% Goat serum and 0.3% TritonX-100 in PBS) for 1 h at room temperature, brain slices were incubated with primary antibodies diluted in blocking buffer overnight at 4 °C. After rinsing in PBS, sections were incubated with secondary antibodies in blocking buffer for 1–2 h at 37 °C, followed by DAPI staining for 15 min at room temperature. Primary antibodies and secondary antibodies are listed in Supplementary Table 1.

### Animal work

All animal experiments were approved by the Animal Ethics Committee of Shanghai Medical College, Fudan University, and carried out in accordance with institutional guidelines. C57BL/6 background mice used in this study were kept under controlled temperature (21–23 °C), and on a standard 12 h light/dark cycle with ad libitum food and water. All of the mice were housed in a specific pathogen-free (SPF) animal facility at the Children’s Hospital of Fudan University. All age and sex-matched mice were randomly subjected to experimental analysis. For animal studies, grouping was performed based on animal genotype with no randomization or blinding used.

### *Wwc1/2* conditional knockout mice

The *Wwc1/2*^*fl/fl*^ conditional knockout mice were described previously [15]. Mice with conditional deletion of *Wwc1/2* in neurons (*Wwc1/2*^*Syn*-cKO^) were generated by first crossing *Wwc1/2*^*fl/fl*^ females with *Syn*-Cre transgenic mice (catalog no.110132, BIOCYTOGEN, Beijing, China). Then, *Wwc1/2*^*Syn*-het^ males were crossed with *Wwc1/2*^*fl/fl*^ to obtain homozygous cKO mice (*Wwc1/2*^*Syn*-cKO^). *Wwc1/2*^*fl/fl*^ mice were used as controls. *Wwc1/2*^*Nes*-cKO^ were generated by crossing *Wwc1/2*^*fl/fl*^ females with *Nestin*-Cre mice (Shanghai Model Organisms Center Inc., Shanghai, China) as described above. The following primers were used for genotyping: *Wwc1* (5’-TGAATATCTCCACTATTGCTCTCGC-3’ and 5’-CCATTCCCTTTCGTCTTCCTC-3’; band sizes for *Wwc1*^*fl/+*^ mice are 218 base pairs (bp) (wild-type allele) and 331 bp (targeted allele with 3’ loxP)); *Wwc2* (5’-CCTTCTGTGTGCTCAGTGGCT-3’ and 5’-AAGGTTCAGTGCTATTGGGAGC-3’; bands sizes for *Wwc2*^*fl/+*^ mice are 307 bp (wild-type allele) and 420 bp (targeted allele with 3’ loxP)); *Syn*-Cre (5’-ATCGGGATCCACATTCGCCTCAGTCTCAGCTTC-3’ and 5’-ATCGCTCGAGAGAGCTCCAGGAGAGGATTCGAT-3’/5’-GCACACAGACAGGAGCATCTTC-3’; band sizes for *Syn*-Cre^Mut/+^ mice are 726 bp (wild-type allele) and 582 bp (mutant-type allele)); *Nestin*-Cre (5’-TTGCTAAAGCGCTACATAGGA-3’ and 5’-GCCTTATTGTGGAAGGACTG-3’/ 5’-CCTTCCTGAAGCAGTAGAGCA-3’; band sizes for *Nestin*-Cre^Mut/+^ mice are 246 bp (wild-type allele) and 150 bp (mutant-type allele)). Genotyping was carried out using standard PCR protocols. For timed pregnancies, the plug date was designated as E0.5, and the date of birth was defined as P0.

### Behavioral tests

All mice used for behavioral tests were 8–10-week-old age-matched male littermates with comparable body weight. All behavioral tests were performed between 9:30 a.m. to 18:00 p.m. All mice (in their home cages) were habituated for 1–2 h in the testing room before any behavioral tests. After each test, the apparatus was cleaned with 75% ethanol. All behavioral assays were done blind to genotypes.

### Open field

Open field test is used for evaluating spontaneous locomotion activity and the anxiety state of animals. The test mouse was gently placed in the corner of the open field apparatus (50 × 50 × 50 cm) in 30 lux and allowed to explore freely for 10 min. The mouse’s activity distance, velocity, and time spent in the central square region (20 × 20 cm) were recorded by overhead video camera and further analyzed with EthoVision XT 14.0 software (Noldus, Holland).

### Elevated plus maze

The elevated plus maze apparatus is constructed of black Plexiglass and elevated about 40 cm above the ground, consisting of two open arms (30 × 6 cm) across from each other, perpendicular to two closed arms (30 × 6 × 16 cm), and a central platform (6 × 6 cm). To assess anxiety, the test mouse was initially positioned in the central platform facing an open arm, and the following 5 min of the video was collected. Total distance and the time spent in the open arms and closed arms were directly measured and analyzed with EthoVision XT 14.0 software (Noldus, Holland).

### Tail suspension

The mouse was suspended by adhesive tape placed ~1 cm from the tip of the tail in the middle of a test cage. After 2 min of accommodation, the total time spent immobile, defined as the absence of struggling and just hanging passively without any movement of the whole body, was recorded during the last 4 min of the test.

### Y maze

Spontaneous alternation of the Y maze is used to measure spatial working memory. The Y maze apparatus, made of black Plexiglass, consists of three opaque plastic arms (30 × 10 × 15 cm, at a 120° angle from each other), designated A, B, and C, and each arm ends with a wall. The mouse was initially placed in the distal end of arm A and allowed to freely explore the maze for 8 min. The movement of each mouse was recorded by the overhead video camera and further analyzed with EthoVision XT 14.0 software (Noldus, Holland). The arm entries were recorded and the percentage of spontaneous alternations (entry into an arm that differs from the previous two entries) was calculated with the following formula: (Alternations/Arm Entries-2) × 100.

### Novel object recognition

The device used for the novel object recognition test was the same one used for the open field test. The test mice were handled before training and acclimated in the test box for 3 days, with 10 min habituation each day. On day 4, two identical objects were placed in the parallel corners of the arena 10 cm from the side walls. The test mouse was placed at the opposite side of the arena and allowed to freely explore the arena for 10 min. After 1 h, one object was randomly replaced with another novel object, which was of the same size but different in color and shape from the familiar one. Then, the same mouse was put back into the same box and allowed to freely explore the two different objects for another 10 min. The interaction time with the familiar and novel object was recorded by video camera above the test box and further analyzed with EthoVision XT 14.0 software (Noldus, Holland).

### Rotarod

The test was carried out using an accelerating rotarod (Med Associates, Vermont, USA) to assess the motor coordination and balance ability of mice. The test consisted of four trials per day for four days, with a minimum of 20 min of recovery time between trials. The rotarod was started initially at 5 rpm and increased to 30 rpm within 5 min. The trial started once the test mouse was loaded into the partitioned compartment. The latency of each mouse to fall from the rotarod was recorded for further analysis.

### Morris Water Maze

The test was performed in a circular tank (120 cm diameter) filled with opaque water (21–23 °C). A 10 cm-circular plexiglass platform submerged 1 cm below the surface of the water was placed at a fixed point at one quadrant. The device with matching software was purchased from Ji Liang Technology Co., Ltd (Shanghai, China). The test mouse was released from four possible starting locations and the order of starting locations was randomly determined. Each trial lasted 1 min and ended when the test mouse successfully climbed onto and remained on the platform for 10 s. The mouse that failed to reach the platform within the 60 s was guided to the platform and stayed for 20 s to remember location information. Four trials per day were conducted for 4 consecutive training days. The time spent by the mouse to reach the platform was recorded as its latency. Time for four trials was averaged and recorded as a result of each mouse. On day 5, the mouse was subjected to a single 60-s probe trial without a hidden platform to test memory retention. The test mouse was released from the distant point opposite the platform. The swimming path and time spent in the target quadrant were recorded automatically.

### Fear conditioning

The test was performed in a fear conditioning apparatus (Med Associates, Vermont, USA; 25 × 30 × 25 cm). For the training phase, the test mouse was placed in the chamber for 6 min followed by 2 min of accumulation, recorded as a baseline, and five repeated pairings of conditional stimulus (auditory tone, 20 s, 90 dB) and unconditional stimulus (3-foot shocks of 0.75 mA during the last 2 s of tone) at a 60 s interval. Following 24 h of training, the test mouse was put back into the same chamber to evaluate the contextual fear memory, and 5 min of recordings were made (context freezing). After 2 h recovery, the test mouse was placed in a new and redecorated chamber (different walls and flooring) and given the conditioned cue (90 dB noise) for 5 min of recordings (cued freezing). Data were analyzed using Med Associates software (MED Associates, St. Albans, VT, USA).

### Viral infection

Newborn pups (P0) were intracerebroventricularly injected with virally-encoded transgenes of AAV2/9-hSyn-AMOT (1.0 × 10^8^ particles/hemisphere) (HANBIO, Shanghai, China). Administer an average volume of 1 μl into each ventricle. Two weeks after viral infection, animals were anesthetized and intracardially perfused with 4% paraformaldehyde as described above, and brains were fixed and sectioned into 30 μm sagittal slices with a vibratome (Leica, VT1000S). Eight to ten weeks after viral infection, animals were used for behavioral tests.

### Golgi staining

For studying the spine density and morphology of neurons, Golgi-Cox impregnation was performed using the FD Rapid GolgiStainTM Kit (PK401FD, NeuroTechnologies, Waltham, Maine, USA), according to the manufacturer’s instructions. In brief, age-matched mice were deeply anesthetized with 0.7% pentobarbital sodium, and brains were quickly removed and immersed into a mixture containing equal volumes of solution A and B at room temperature for 2 weeks. Then, the brains were transferred into solution C for at least 72 h. Serial coronal vibratome sections (130 μm) through the entire brain were mounted onto the gelatin-coated microscope slides (PO101FD, NeuroTechnologies) and stained using solutions D and E followed by image analysis.

### Image acquisition and analysis

Confocal images were acquired using Zeiss LSM 880 with Airyscan with a ×10 objective at 1024 × 1024 pixel resolution. For dendritic spine density analysis, images were collected by Leica TCS SP8 with ×63/1.4 NA oil immersion objective at 3× optical zoom and 0.3 μm Z-interval. The spine density was analyzed with ImageJ (Fiji, Rawak Software Inc., Stuttgart, Germany) software blinded to the genotype.

### Statistical analysis

All data quantifications are presented as the mean ± SD of at least three independent experiments. Results were analyzed using Prism 8.0 software (GraphPad, SanDiego, CA, USA). Statistical significance was determined using Student’s *t* test or one-way ANOVA between groups. *p < 0.05, **p < 0.01, ***p < 0.001, ****p < 0.0001, n.s indicates not significant.

## Supplementary information

Figure S1

Figure S2

Figure S3

Figure S4

Figure S5

Figure S6

SUPPLEMENTAL MATERIALS

Manuscript-clean

Confirmation of authorship

Original western blots

aj-Checklist

## Data Availability

The data of this study are available from the corresponding author upon reasonable request.
