# Supplementary figures and images for: WWC1/2 regulate spinogenesis and cognition in mice by stabilizing AMOT
Source: Cell Death Dis. 2023 Aug 1;14(8):491. doi: 10.1038/s41419-023-06020-7 (PMC10394084; doi:10.1038/s41419-023-06020-7)

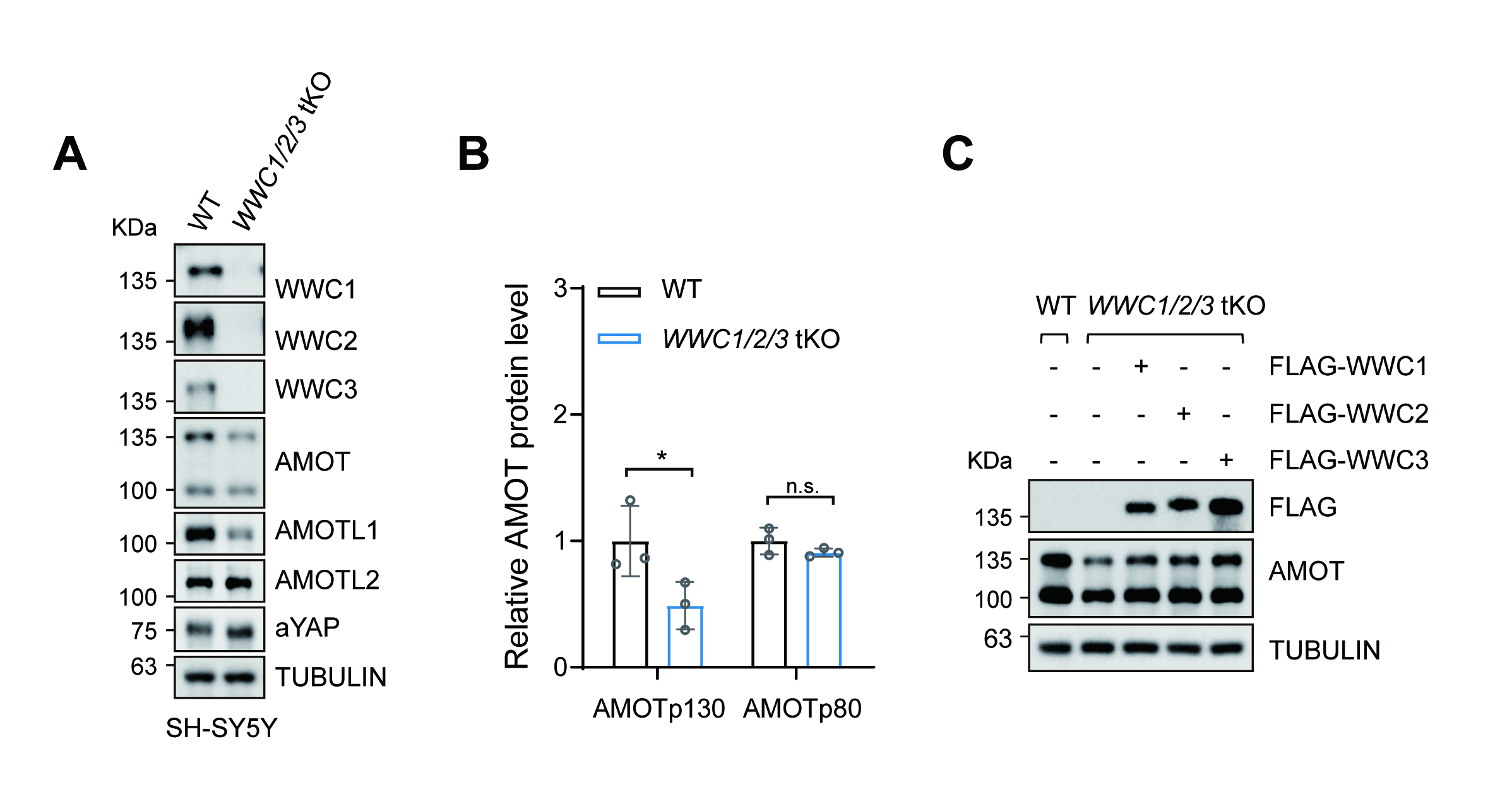

Supplement: Supplementary file 1 — Figure S1 [file 41419_2023_6020_MOESM1_ESM.tif]

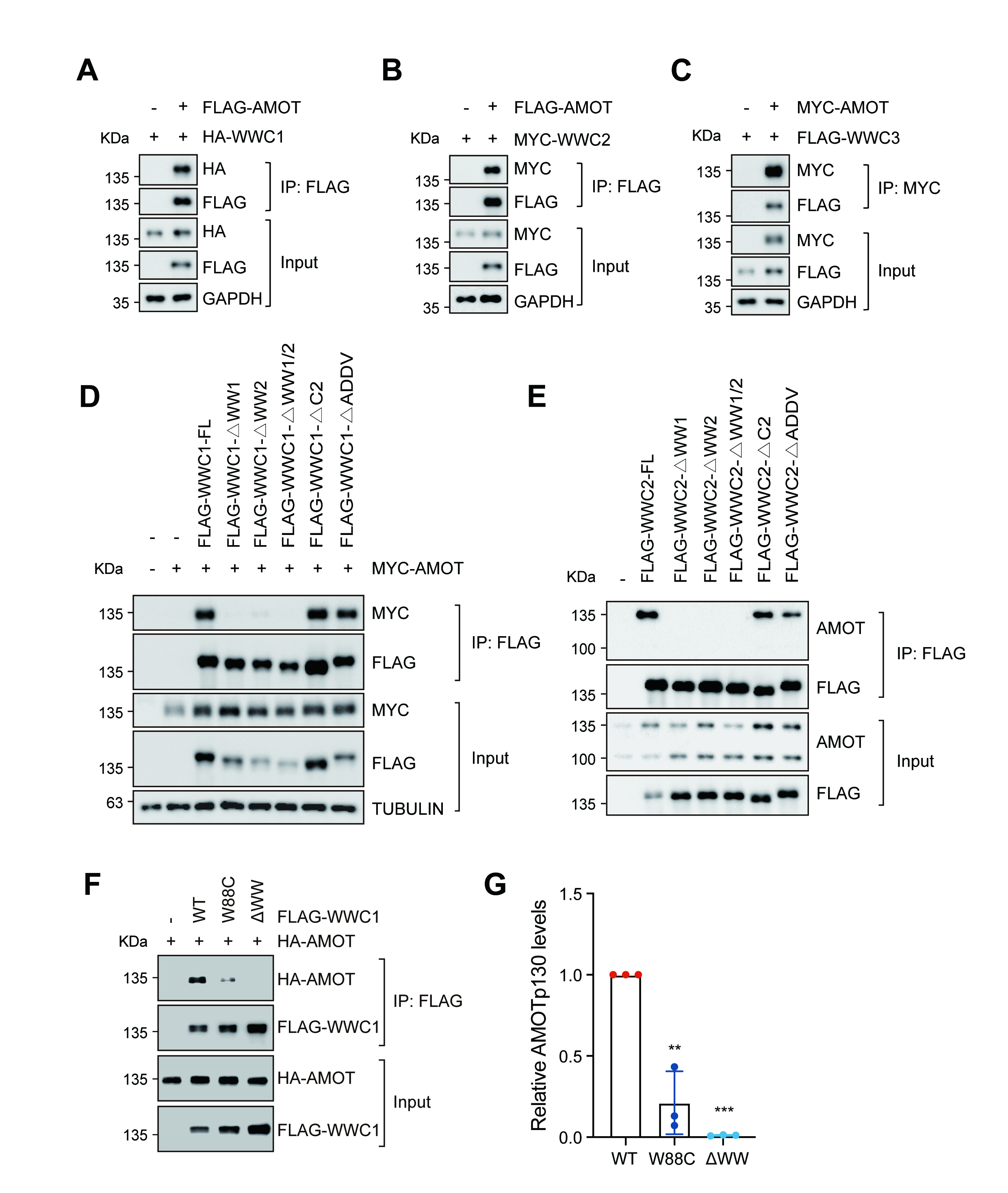

Supplement: Supplementary file 2 — Figure S2 [file 41419_2023_6020_MOESM2_ESM.tif]

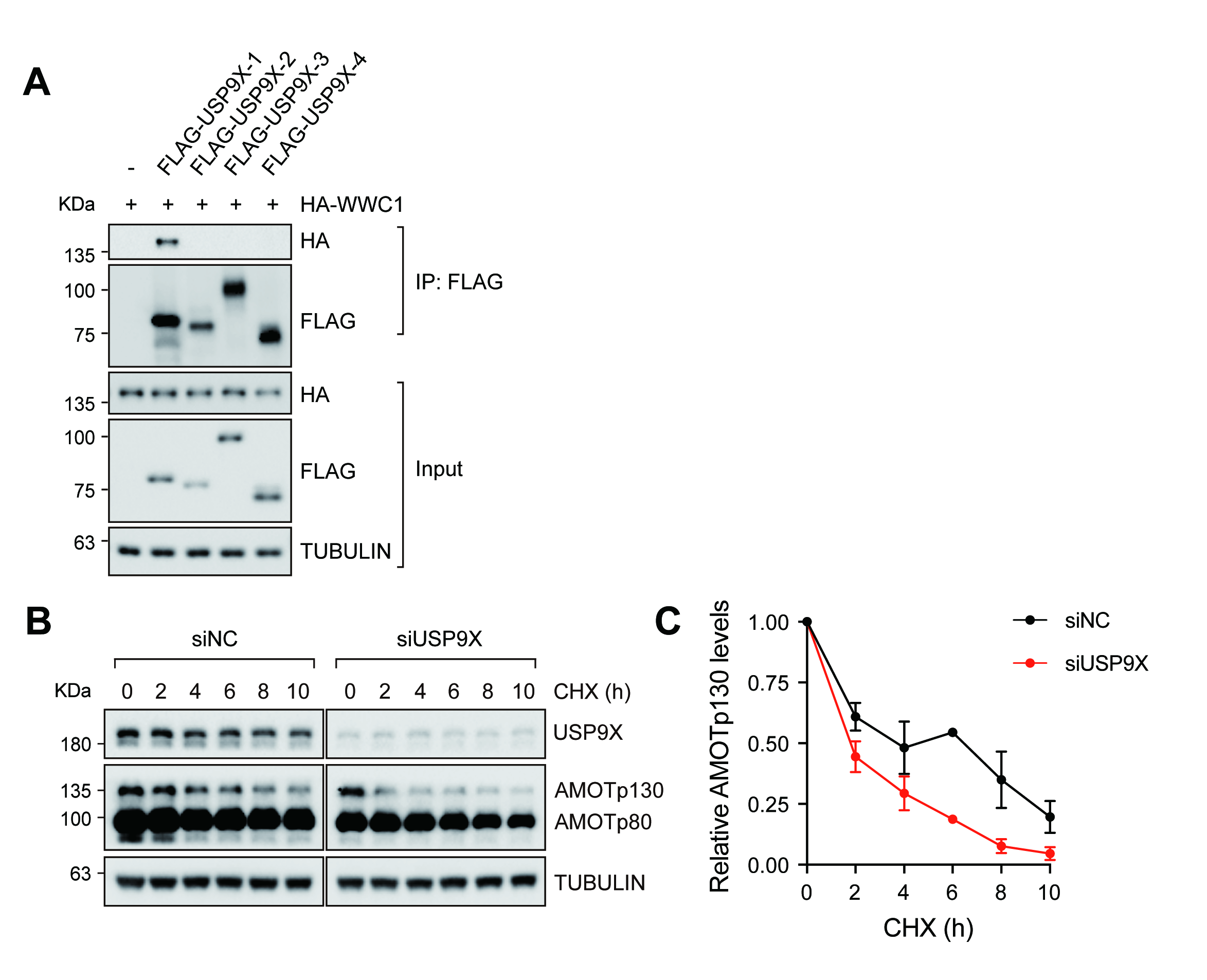

Supplement: Supplementary file 3 — Figure S3 [file 41419_2023_6020_MOESM3_ESM.tif]

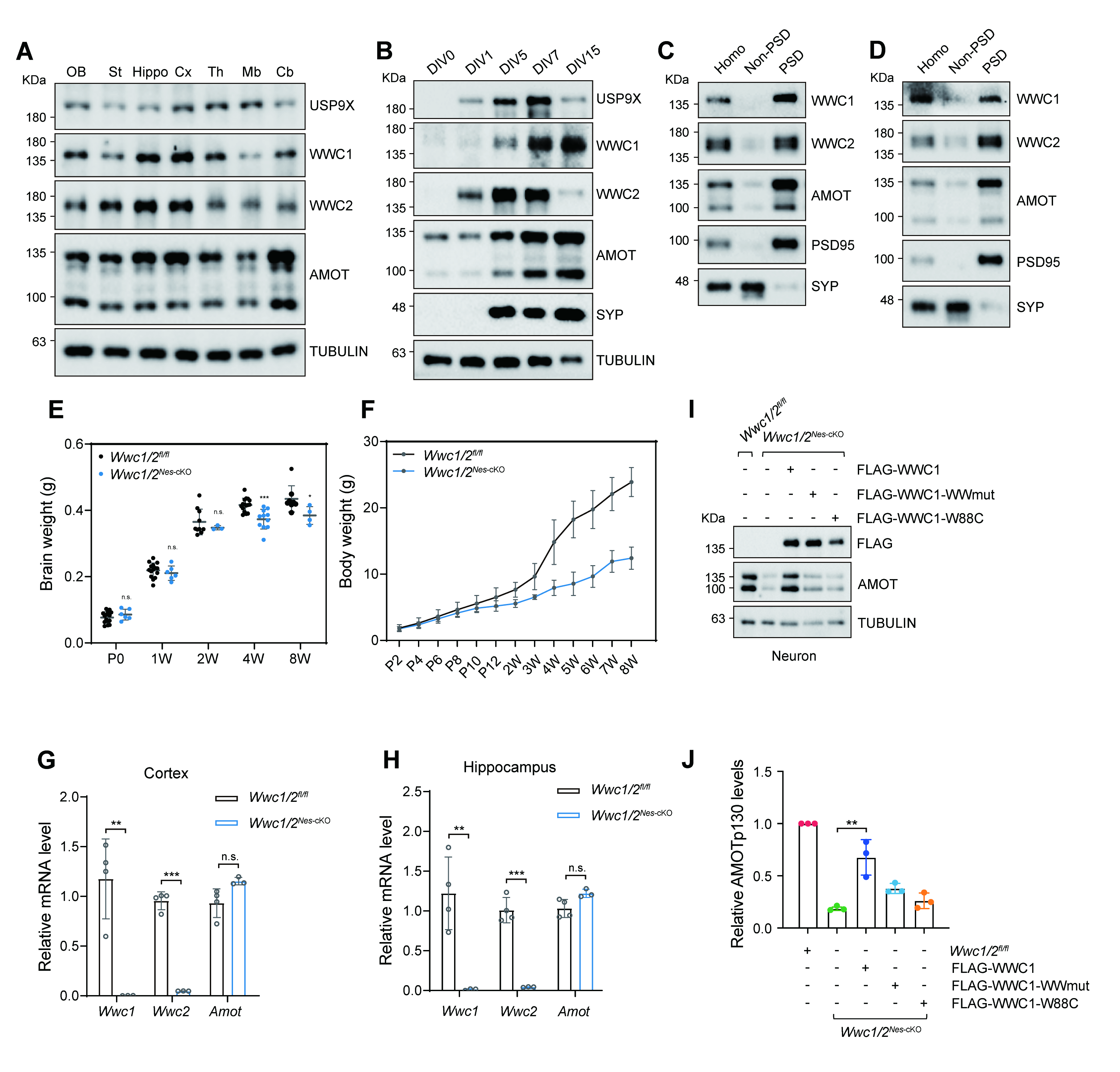

Supplement: Supplementary file 4 — Figure S4 [file 41419_2023_6020_MOESM4_ESM.tif]

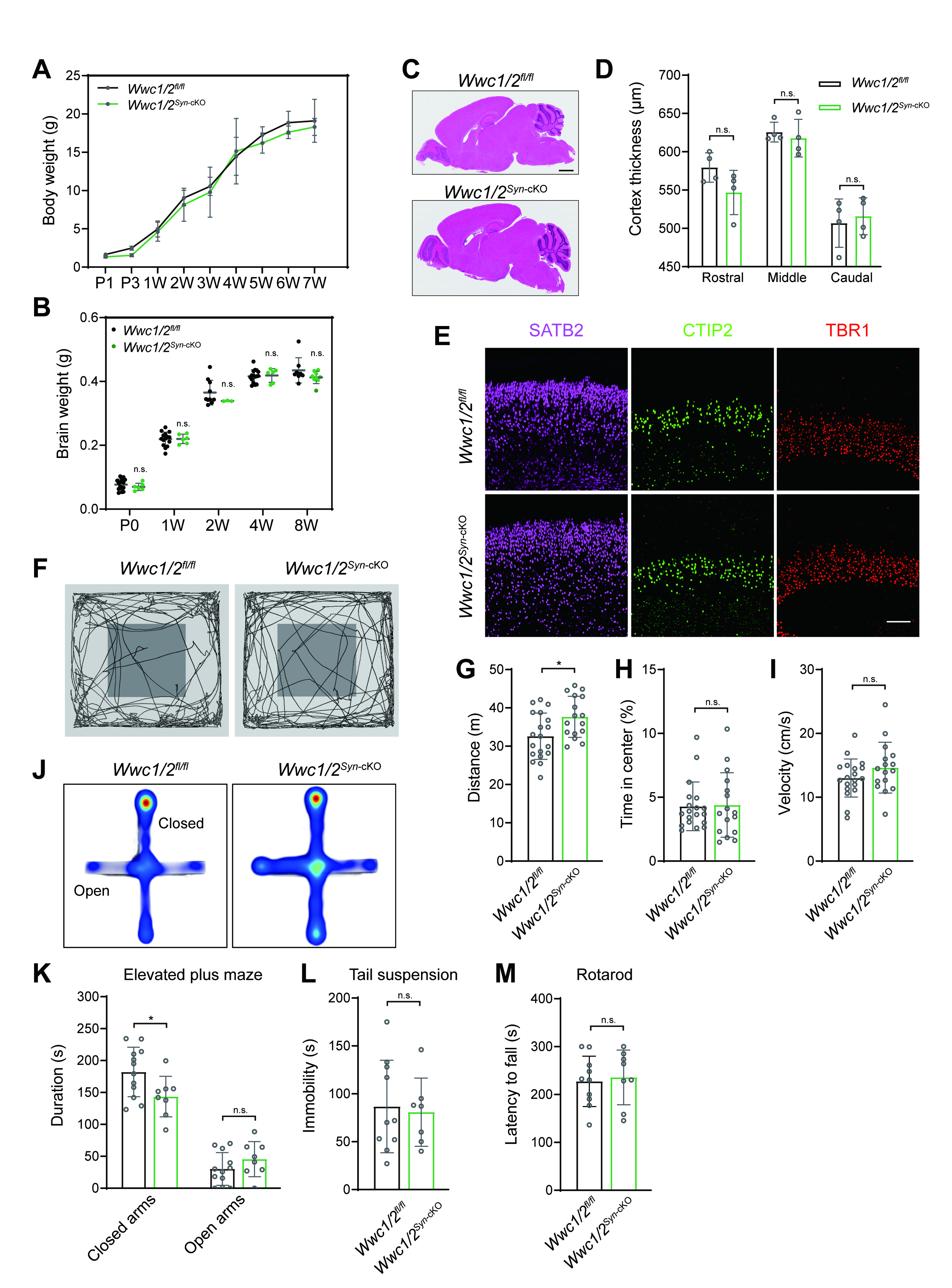

Supplement: Supplementary file 5 — Figure S5 [file 41419_2023_6020_MOESM5_ESM.tif]

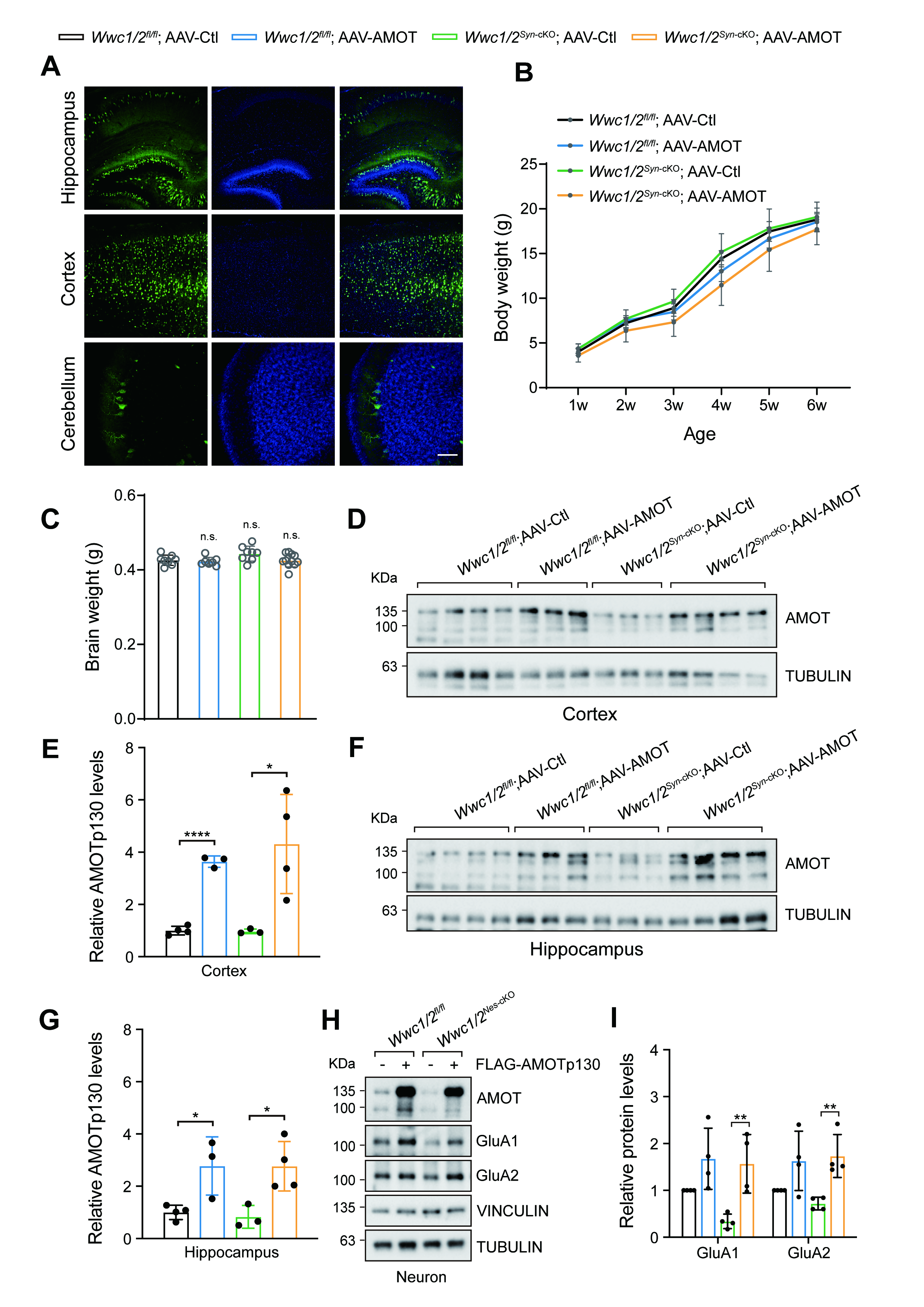

Supplement: Supplementary file 6 — Figure S6 [file 41419_2023_6020_MOESM6_ESM.tif]

Figure 1

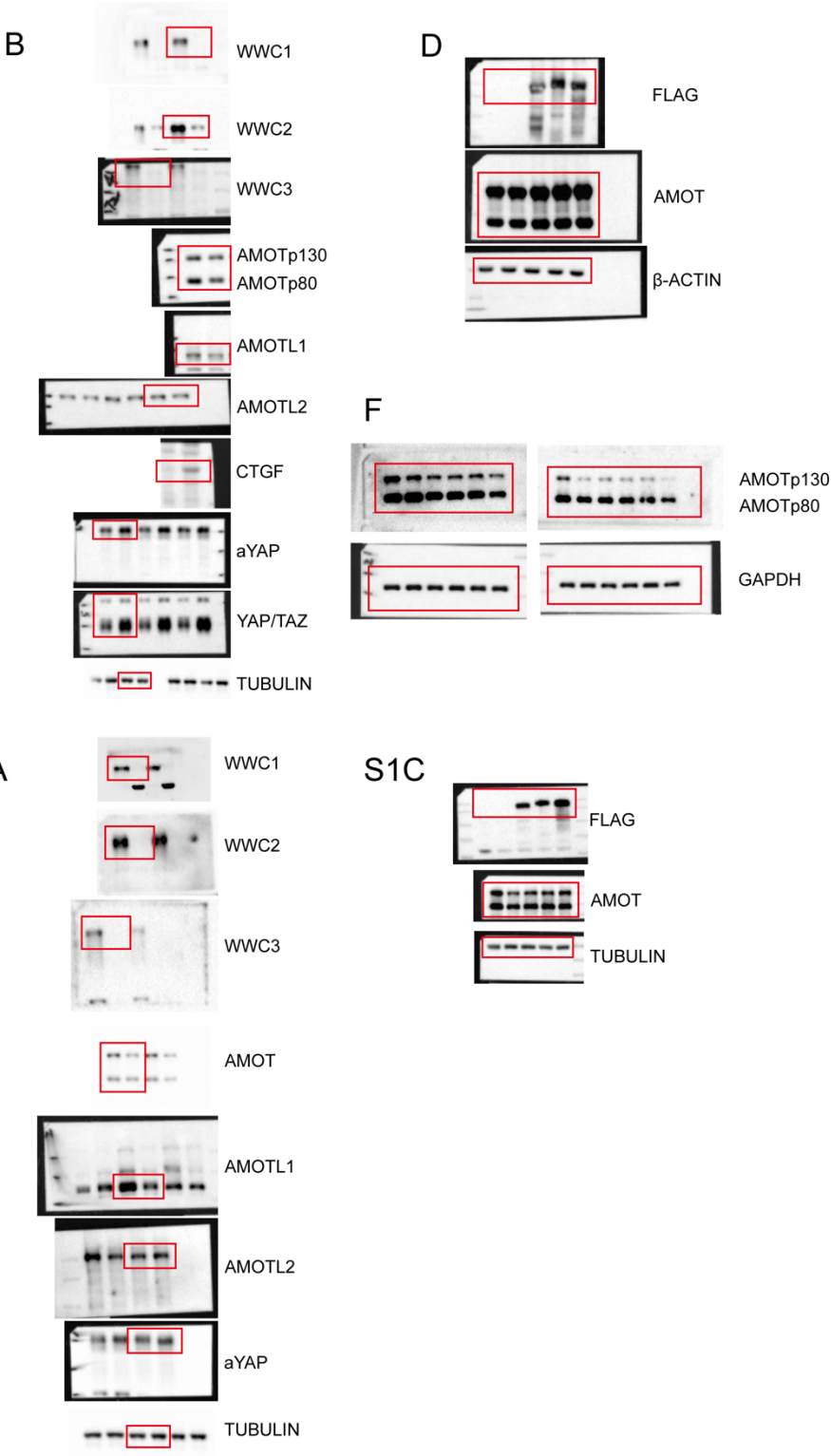

Figure 2

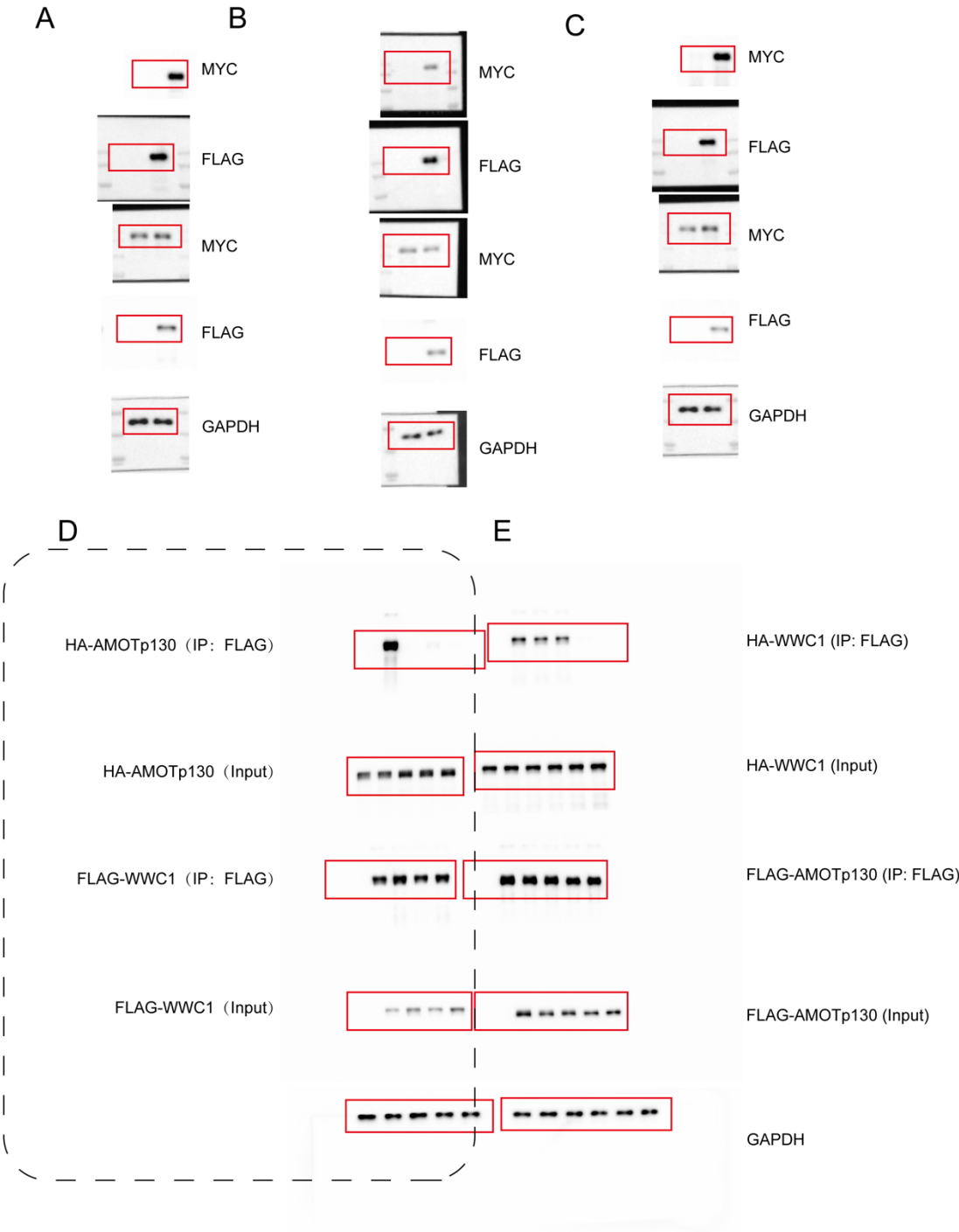

Figure 2

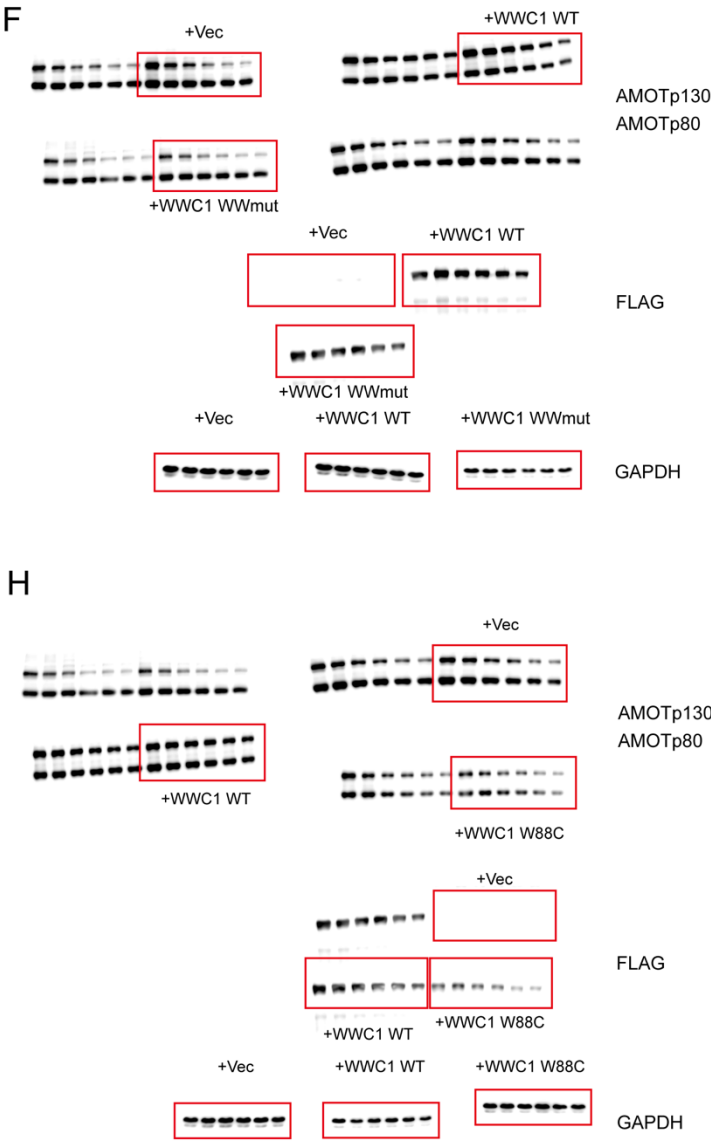

Figure S2

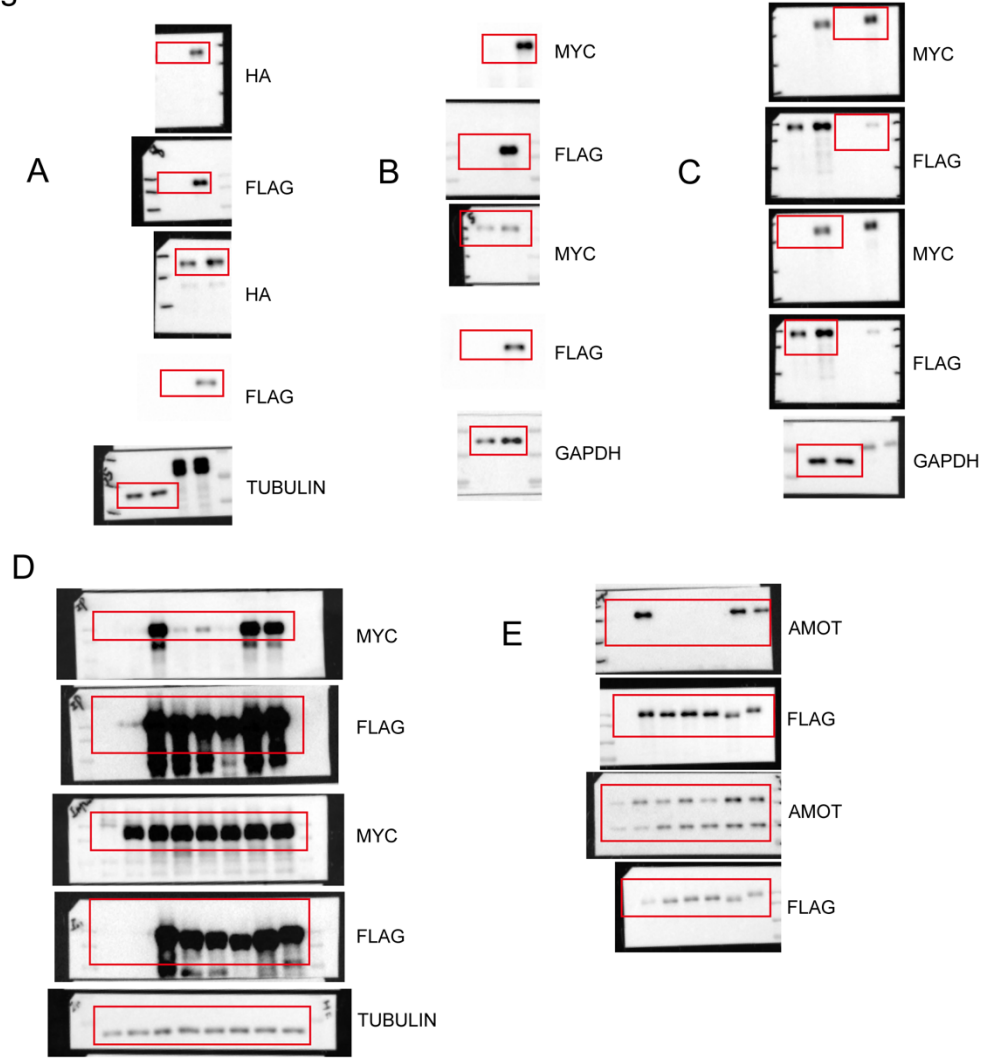

Figure S2

F

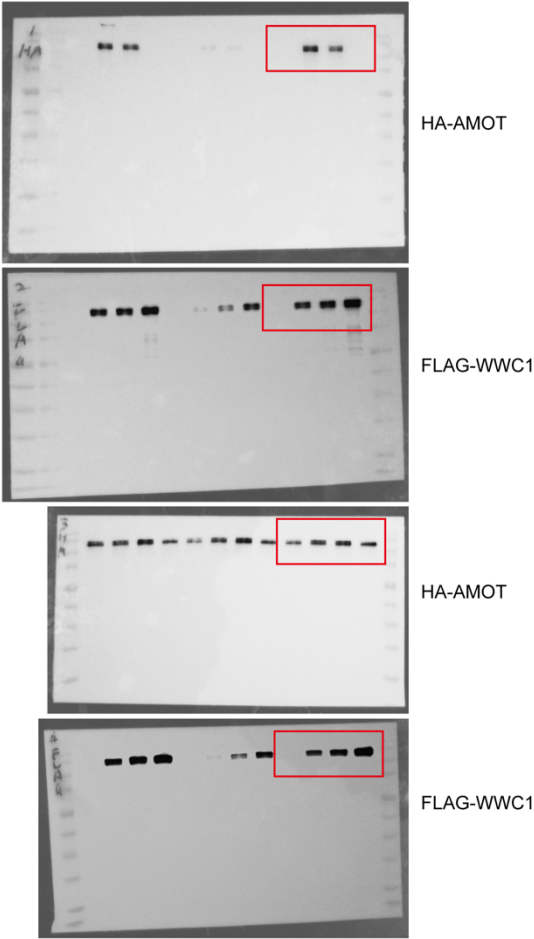

Figure 3

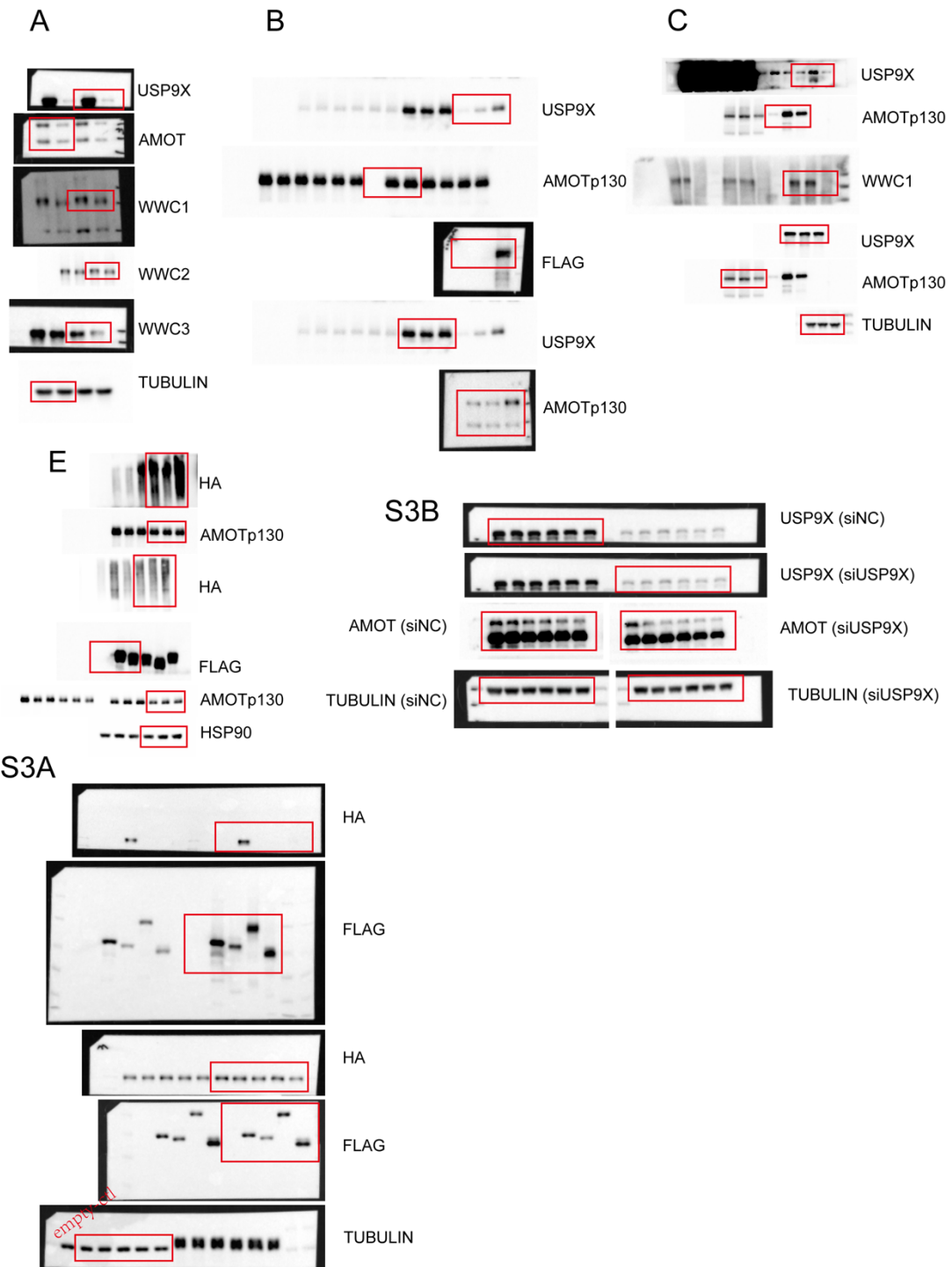

Figure 3

D

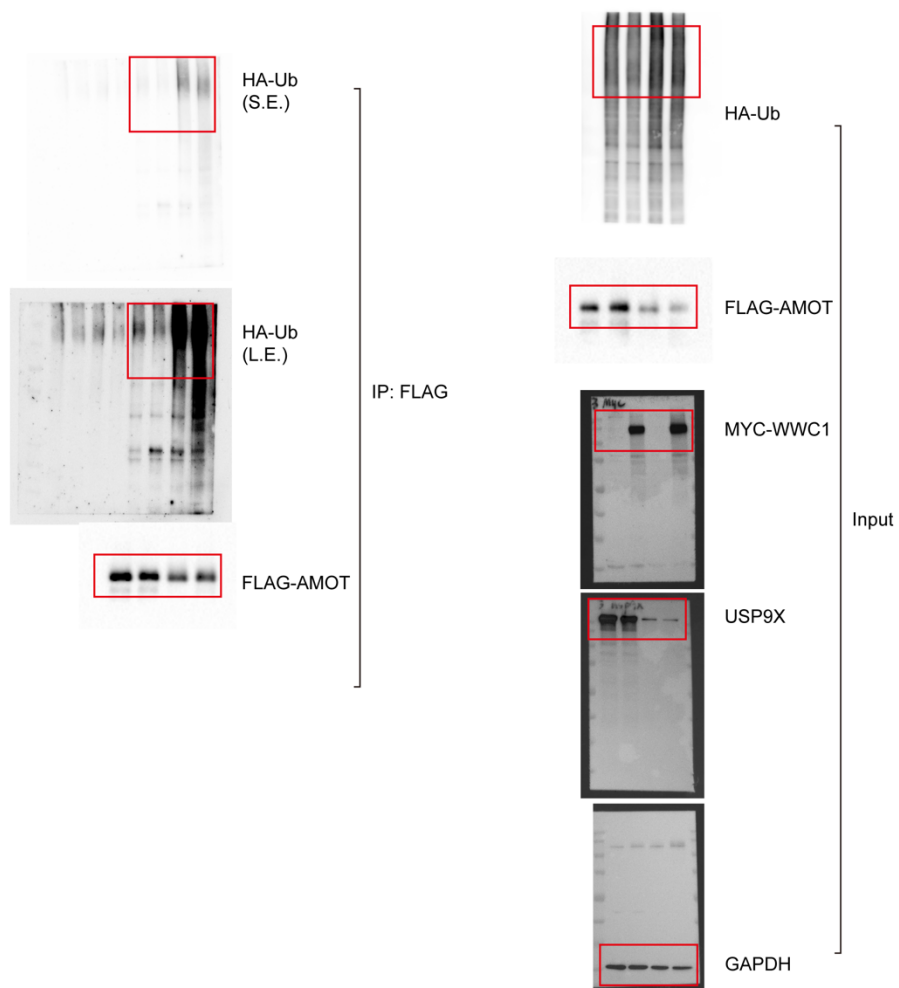

Figure 4

A

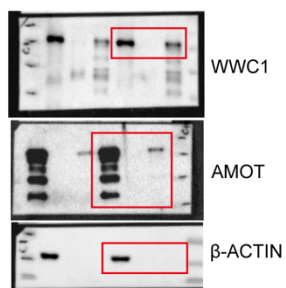

D

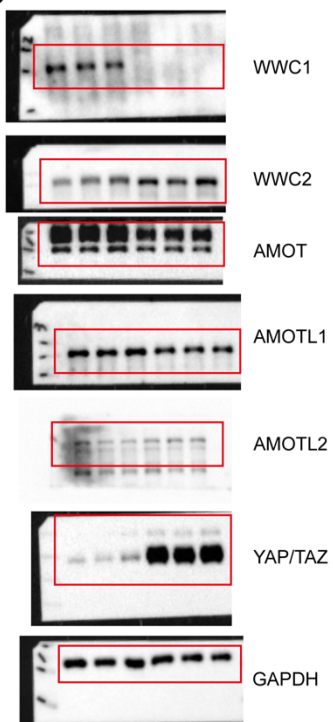

F

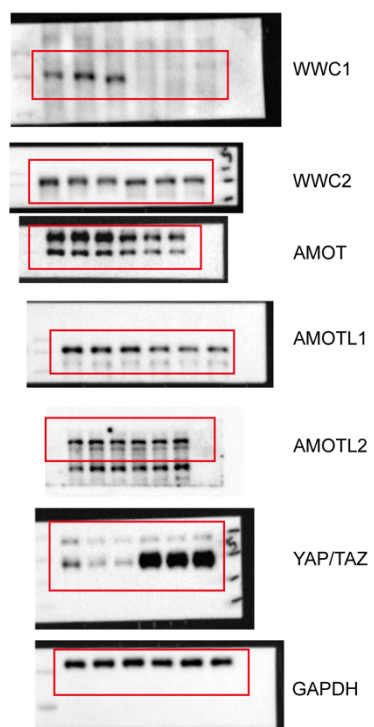

H

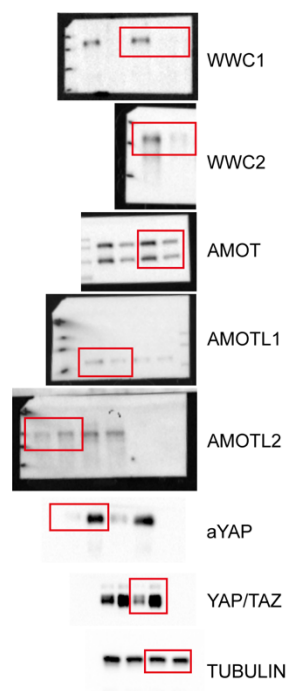

Figure S4

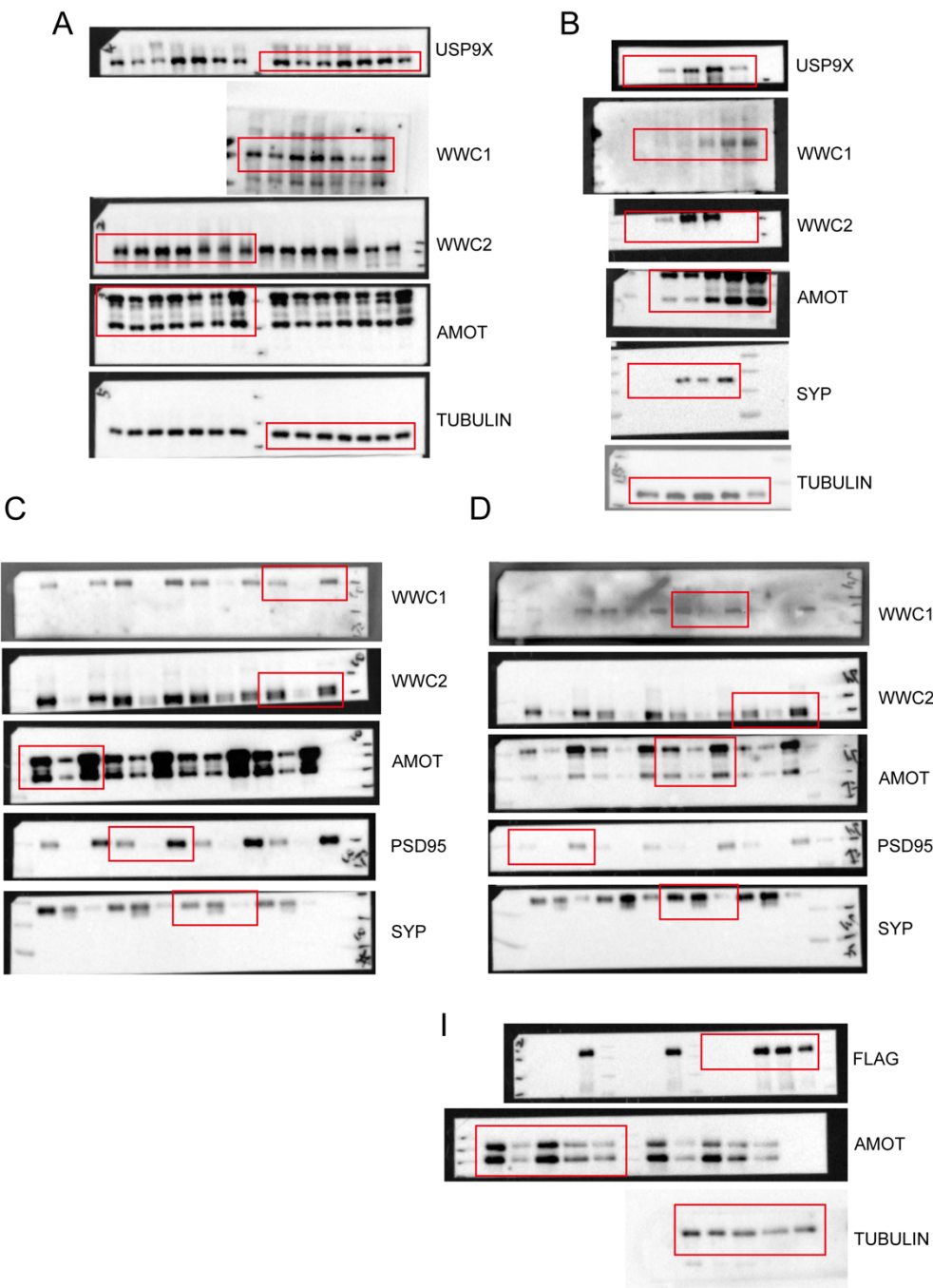

Figure S6

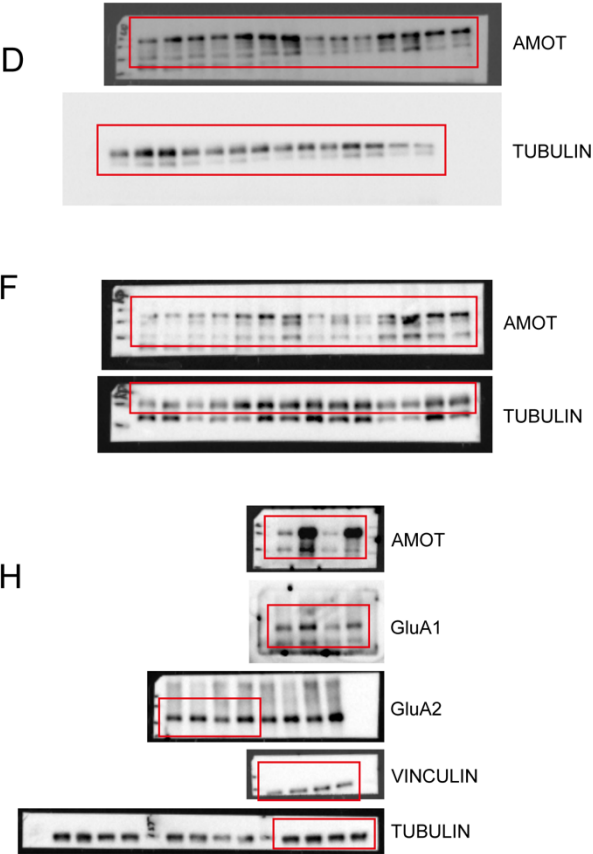

Supplement: Supplementary file 10 — Original western blots [file 41419_2023_6020_MOESM10_ESM.pdf]
