# Supplementary material for: WWC1/2 regulate spinogenesis and cognition in mice by stabilizing AMOT
Source: Cell Death Dis. 2023 Aug 1;14(8):491. doi: 10.1038/s41419-023-06020-7 (PMC10394084; doi:10.1038/s41419-023-06020-7)
Supplement: Supplementary file 7 — SUPPLEMENTAL MATERIALS [file 41419_2023_6020_MOESM7_ESM.docx]

**SUPPLEMENTAL MATERIALS**

**WWC1/2 regulate spinogenesis and cognition in mice by stabilizing AMOT**

Runyi Cao^1,#^, Rui Zhu^1,#^, Zhao Sha^1^, Sixian Qi^1^, Zhenxing Zhong^1^, Fengyun Zheng^1^, Yubin Lei^1^, Yanfeng Tan^1^, Yuwen Zhu^1^, Yu Wang^1,*^, Yi Wang^2,*^, Fa-Xing Yu^1,*^

^1^Institute of Pediatrics, Children's Hospital of Fudan University, and the Shanghai Key Laboratory of Medical Epigenetics, The International Co-laboratory of Medical Epigenetics and Metabolism, the State Key Laboratory of Genetic Engineering, Institutes of Biomedical Sciences, Shanghai Medical College, Fudan University, Shanghai 200032, China.

^2^Department of Neurology, Children's Hospital of Fudan University, National Children's Medical Center, No. 399 Wanyuan Road, Shanghai 201102, China

^#^These authors contributed equally

*To whom correspondence should be addressed. E-mail: fd_wangyu@fudan.edu.cn (YuW), yiwang@shmu.edu.cn (YiW), and fxyu@fudan.edu.cn (FXY)

**SUPPLEMENTAL TABLE**

**Table S1. KEY RESOUECE TABLE**

| REAGENT or RESOURCE | SOURCE | IDENTIFIER |
| --- | --- | --- |
| Antibodies | | |
| Rabbit polyclonal anti-Angiomotin | Bethyl Laboratories | Cat#A303-305A; RRID: AB_10951678 |
| Mouse monoclonal anti-HA tag (F-7) | Santa Cruz Biotechnology | Cat#sc-7392; RRID: AB_627809 |
| Goat polyclonal anti-CTGF (L-20) | Santa Cruz Biotechnology | Cat#sc-14939; RRID: AB_638805 |
| Rabbit monoclonal anti-HA Tag (C29F4) (HRP Conjugate) | Cell Signaling Technology | Cat#14031S; RRID: AB_2798368 |
| Rabbit monoclonal anti-YAP (D8H1X) | Cell Signaling Technology | Cat#14074S; RRID: AB_2650491 |
| Rabbit monoclonal anti-YAP/TAZ (D24E4) | Cell Signaling Technology | Cat#8418S; RRID: AB_10950494 |
| Rabbit monoclonal anti-AMPA Receptor 1 (GluA1) (D4N9V) | Cell Signaling Technology | Cat#13185S; RRID: AB_2732897 |
| Rabbit monoclonal anti-AMPA Receptor 2 (GluA2) (E1L8U) | Cell Signaling Technology | Cat#13607S |
| Rabbit monoclonal anti-Vinculin (E1E9V) | Cell Signaling Technology | Cat#13901S; RRID: AB_2728768 |
| Mouse monoclonal anti-α-Tubulin | Sigma-Aldrich | Cat#T6199-100UL; RRID: AB_477583 |
| Rabbit polyclonal anti-AMOTL1 | Sigma-Aldrich | Cat#HPA001196; RRID: AB_1078147 |
| Mouse monoclonal anti-FLAG Tag (HRP conjugated) | Sigma-Aldrich | Cat#A8592; RRID: AB_439702 |
| Mouse monoclonal anti-HSP90 | BD Bioscience | Cat#610418; RRID: AB_397798 |
| Rabbit polyclonal anti-AMOTL2 antibody | Abcam | Cat#ab135722 |
| Rat anti-CTIP2 | Abcam | Cat#ab18465; RRID: AB_2064130 |
| Goat polyclonal anti-PSD95 | Abcam | Cat#ab12093; RRID: AB_298846 |
| Rabbit monoclonal anti-SATB2 | Abcam | Cat#ab92446; RRID: AB_10563678 |
| Rabbit polyclonal anti-WWC2 | Abcam | Cat#ab126356; RRID: AB_11140331 |
| Rabbit monoclonal anti-active YAP1 (EPR19812) | Abcam | Cat#ab205270; RRID: AB_2813833 |
| Mouse monoclonal anti-MYC Tag | Proteintech | Cat#60003-2-Ig; RRID: AB_2734122 |
| Mouse monoclonal anti-TBR1 | Proteintech | Cat#66564-1-Ig; RRID: AB_2881925 |
| Rabbit Polyclonal anti-Synaptophysin | Proteintech | Cat#17785-1-AP; RRID: AB_2271365 |
| HRP-conjugated Affinipure Rabbit Anti-Goat IgG(H+L) | Proteintech | Cat#SA00001-4; RRID: AB_2864335 |
| Mouse monoclonal anti-GAPDH (HRP conjugated) | Abways Technology | Cat#AB2000 |
| Peroxidase AffiniPure Goat Anti-Mouse IgG (H+L) | Jackson ImmunoResearch Laboratories | Cat#115-035-003; RRID: AB_10015289 |
| Peroxidase AffiniPure Goat Anti-Rabbit IgG (H+L) | Jackson ImmunoResearch Laboratories | Cat#111-035-003; RRID: AB_2313567 |
| Alexa Fluor® 647-AffiniPure Goat Anti-Rabbit IgG (H+L) | Jackson ImmunoResearch Laboratories | Cat#111-605-045; RRID: AB_2338075 |
| Alexa Fluor® 488-AffiniPure Goat Anti-Rat IgG (H+L) | Jackson ImmunoResearch Laboratories | Cat#112-545-003; RRID: AB_2338351 |
| Goat anti-rabbit IgG antibody, Alexa Fluor 488 | Thermo Fisher Scientific | Cat#A11008; RRID: AB_143165 |
| Goat anti-mouse IgG antibody, Alexa Fluor 555 | Thermo Fisher Scientific | Cat#A21422; RRID: AB_2535844 |
| Goat anti-rabbit IgG antibody, Alexa Fluor 555 | Thermo Fisher Scientific | Cat#A21428; RRID: AB_2535849 |
| Goat anti-mouse IgG antibody, Alexa Fluor 488 | Thermo Fisher Scientific | Cat#A11001; RRID: AB_2534069 |
| Mouse monoclonal anti-WWC1 | PMID: 22904328 | N/A |
| Rabbit polyclonal anti-WWC3 | This paper | N/A |
| Bacterial and virus strains |  |  |
| *Trans*5α Chemically Competent Cell | TransGen Biotech | Cat#CD201-01 |
| PLVX lentivirus | This paper | N/A |
| Adeno-associated viruses | HANBIO | N/A |
| Chemicals | | |
| B-27 | Thermo Fisher Scientific | Cat#17504044 |
| DAPI | Sigma-Aldrich | Cat#D9542 |
| Fetal bovine serum | Invitrogen | Cat#10091-148 |
| Goat serum | Gibco | Cat#16210-064 |
| Protease inhibitor cocktail | MCE | Cat#HY-K0010 |
| Phosphatase Inhibitor Cocktail Ⅰ | MCE | Cat#HY-K0021 |
| Phosphatase Inhibitor Cocktail Ⅱ | MCE | Cat#HY-K0022 |
| Protein A/G Plus-Agarose | SantaCruz | Cat#sc-2003 |
| FLAG-beads | SMART Lifesciences | SA042005 |
| PolyJet | Signagen Laboratories | Cat#SL100688 |
| Prime STAR Max DNA Polymerase | Takara | Cat#R045A |
| T4 ligase | Takara | Cat#2011A |
| Critical commercial assays | | |
| TaKaRa MiniBEST Universal RNA Extraction Kit | Takara | Cat#9767 |
| TB Green® *Premix* *Ex Taq™*(Tli RNaseH Plus) | Takara | Cat#RR420A |
| *TransScript*® First-Strand cDNA Synthesis SuperMix | TransGen Biotech | Cat#AT301-03 |
| Gibson Assembly® Master Mix | NEB | Cat#E2611L |
| ClonExpress MultiS One Step Cloning Kit | Vazyme | Cat#C113-02 |
| High-sig ECL Western Blotting Substrate | Tanon | Cat#180-501 |
| Experimental models: Cell lines | | |
| Human: HEK293A | PMID: 22863277 | N/A |
| Human: HEK293T | National collection of Authenticated Cell Cultures | Cat#SCSP-502 |
| Human: SH-SY5Y | National collection of Authenticated Cell Cultures | Cat#SCSP-5014 |
| HEK293A *WWC1/2/*3 KO | PMID: 35429439 | N/A |
| HEK293A *USP9X* KD | This paper | N/A |
| SH-SY5Y *WWC1/2/3* KO | This paper | N/A |
| Experimental models: Organisms/strains | | |
| Mouse: *Wwc1^fl/fl^*: C57/BL6J-*Wwc1* fl/fl | PMID: 35429439 | N/A |
| Mouse: *Wwc2^fl/fl^*: C57/BL6J-*Wwc2* fl/fl | PMID: 35429439 | N/A |
| Mouse: *Nes-cre*: C57/BL6J-*Nestin cre* | The Jackson Laboratory | Cat#003771 |
| Mouse: *Syn-cre*: C57/BL6J-*Synapsin cre* | BIOCYTOGEN | Cat#110132 |
| Oligonucleotides | | |
| *WWC1* sgRNA#1  5’-GGAAAAGCAAGATCTCATTA-3’ | PMID: 35429439 | N/A |
| *WWC1* sgRNA#2  5’-GTGAAGGGCTGGATAGGACA-3’ | PMID: 35429439 | N/A |
| *WWC2* sgRNA#1  5’-GATCTCCACTACAAGATTAA-3’ | PMID: 35429439 | N/A |
| *WWC2* sgRNA#2  5’-GCTCTGTCAGTAAACACCGA-3’ | PMID: 35429439 | N/A |
| *WWC3* sgRNA#1  5’-TAAGCAGCAGCGGTTCGAGC-3’ | PMID: 35429439 | N/A |
| *WWC3* sgRNA#2  5’-CTACAAACTGGATGAGGCGC-3’ | PMID: 35429439 | N/A |
| *USP9X* shRNA#1  5’-GAGAGTTTATTCACTGTCTTA-3’ | PMID: 29183995 | N/A |
| *USP9X* shRNA#2  5’-CGCCTGATTCTTCCAATGAAA-3’ | PMID: 29183995 | N/A |
| *USP9X* siRNA#1  5’-GAGTGGCTGGAAGTTTGAAATTCAT-3’ | This paper | N/A |
| *USP9X* siRNA#2  5’-CAGACTTAGGTAGCAGCCTAAATAT-3’ | This paper | N/A |
| *USP9X* siRNA#3  5’-GAGATGGAGCAAGAGTACTTATGAA-3’ | This paper | N/A |
| RT-qPCR primer *Wwc1* forward  5’-GTACACCAAACCACTCACCTTCGCT-3’ | This paper | N/A |
| RT-qPCR primer *Wwc1* reverse  5’-TGGTATTGTGGTCTATGAAGTAATC-3’ | This paper | N/A |
| RT-qPCR primer *Wwc2* forward  5’-GTTAACGAAGCCCCTGTCCTTCGCT-3’ | This paper | N/A |
| RT-qPCR primer *Wwc2* reverse  5’-TGTTGATGTGGTCGATGTAGTAGGC-3’ | This paper | N/A |
| RT-qPCR primer *Amot* forward  5’-CCGCCAGAATACCCTTTCAAG-3’ | This paper | N/A |
| RT-qPCR primer *Amot* reverse  5’-CTCATCAGTTGCCCCTCTGT-3’ | This paper | N/A |
| RT-qPCR primer *WWC1* forward  5’-AGCTCCAAGTATGACCCTGAG-3’ | This paper | N/A |
| RT-qPCR primer *WWC1* reverse  5’-AAAGCCACGCTCTTTGAACTG-3’ | This paper | N/A |
| RT-qPCR primer *WWC2* forward  5’-GTCATTTGCTGATTGTGTTGGG-3’ | This paper | N/A |
| RT-qPCR primer *WWC2* reverse  5’-TGGATCTTCTATCTGCGTGGTT-3’ | This paper | N/A |
| RT-qPCR primer *WWC3* forward  5’-CAAGAGCGCATGTTGAAGGAA-3’ | This paper | N/A |
| RT-qPCR primer *WWC3* reverse  5’-CGCTGCTGCTTAATCTGGTAGA-3’ | This paper | N/A |
| RT-qPCR primer *CYR61* forward  5’-AGCCTCGCATCCTATACAACC-3’ | PMID: 22863277 | N/A |
| RT-qPCR primer *CYR61* reverse  5’-TTCTTTCACAAGGCGGCACTC-3’ | PMID: 22863277 | N/A |
| RT-qPCR primer *CTGF* forward  5’-CCAATGACAACGCCTCCTG-3’ | PMID: 22863277 | N/A |
| RT-qPCR primer *CTGF* reverse  5’-TGGTGCAGCCAGAAAGCTC-3’ | PMID: 22863277 | N/A |
| RT-qPCR primer *ANKRD1* forward  5’-CACTTCTAGCCCACCCTGTGA-3’ | PMID: 22863277 | N/A |
| RT-qPCR primer *ANKRD1* reverse  5’-CCACAGGTTCCGTAATGATTT-3’ | PMID: 22863277 | N/A |
| RT-qPCR primer *AMOT* forward  5’-GTATCAGCATCCCCCTGA-3’ | PMID: 34433060 | N/A |
| RT-qPCR primer *AMOT* reverse  5’-GACAATGGCAATGAGATGTCC-3’ | PMID: 34433060 | N/A |
| RT-qPCR primer *AMOTL1* forward  5’-GAACTAGCCATGATCGCCTC-3’ | PMID: 26456820 | N/A |
| RT-qPCR primer *AMOTL1* reverse  5’-ACCTGGACAGGACTACTGGG-3’ | PMID: 26456820 | N/A |
| RT-qPCR primer *AMOTL2* forward  5’-AGCTTCAATGAGGGTCTGCT-3’ | PMID: 26456820 | N/A |
| RT-qPCR primer *AMOTL2* reverse  5’-TGAAGGACCTTGATCACTGC-3’ | PMID: 26456820 | N/A |
| RT-qPCR primer *β-ACTIN* forward  5’-GCCGACAGGATGCAGAAGGAGATCA-3’ | PMID: 22863277 | N/A |
| RT-qPCR primer *β-ACTIN* reverse  5’-AAGCATTTGCGGTGGACGATGGA-3’ | PMID: 22863277 | N/A |
| Recombinant DNA | | |
| PLVXyu-2FLAG-AMOTp130 | PMID: 34433060 | N/A |
| PLVXyu-2FLAG-AMOTp130-LPTY106LATA | This paper | N/A |
| PLVXyu-2FLAG-AMOTp130-PPEY239PAEA | This paper | N/A |
| PLVXyu-2FLAG-AMOTp130-PPEY284PAEA | This paper | N/A |
| PLVXyu-2FLAG-AMOTp130-PPEY239/284PAEA | This paper | N/A |
| PLVXyu-2HA-AMOTp130 | This paper | N/A |
| PLVXyu-2MYC-AMOTp130 | This paper | N/A |
| PLVXyu-2FLAG-Amotp130 | This paper | N/A |
| PLVXyu-2FLAG-WWC1 | PMID: 35429439 | N/A |
| PLVXyu-2HA-WWC1 | PMID: 35429439 | N/A |
| PLVXyu-2MYC-WWC1 | PMID: 35429439 | N/A |
| PLVXyu-2FLAG-WWC2 | This paper | N/A |
| PLVXyu-2HA-WWC2 | This paper | N/A |
| PLVXyu-2MYC-WWC2 | This paper | N/A |
| PLVXyu-2FLAG-WWC3 | This paper | N/A |
| PLVXyu-2MYC-WWC3 | This paper | N/A |
| PLVXyu-2FLAG-WWC1-△WW1 | This paper | N/A |
| PLVXyu-2FLAG-WWC1-△WW2 | This paper | N/A |
| PLVXyu-2FLAG-WWC1-△WW1/2 | This paper | N/A |
| PLVXyu-2FLAG-WWC1-△ADDV | This paper | N/A |
| PLVXyu-2FLAG-WWC1-△C2 | This paper | N/A |
| PLVXyu-2FLAG-WWC1-WW1 mut | PMID: 35429439 |  |
| PLVXyu-2FLAG-WWC1-WW2 mut | PMID: 35429439 |  |
| PLVXyu-2FLAG-WWC1-WW1/2 mut | PMID: 35429439 |  |
| PLVXyu-2FLAG-WWC1-W88C | This paper |  |
| PLVXyu-2FLAG-WWC2-△WW1 | This paper | N/A |
| PLVXyu-2FLAG-WWC2-△WW2 | This paper | N/A |
| PLVXyu-2FLAG-WWC2-△WW1/2 | This paper | N/A |
| PLVXyu-2FLAG-WWC2-△ADDV | This paper | N/A |
| PLVXyu-2FLAG-WWC2-△C2 | This paper | N/A |
| PLVXyu-2FLAG-USP9X-1 (1-600 AA) | This paper | N/A |
| PLVXyu-2FLAG-USP9X-2 (601-1200 AA) | This paper | N/A |
| PLVXyu-2FLAG-USP9X-3 (1201-2000 AA) | This paper | N/A |
| PLVXyu-2FLAG-USP9X-4 (2001-2555 AA) | This paper | N/A |
| pCDNA-HA-Ub | This paper | N/A |
| Software and algorithms | | |
| GraphPad Prism 8 | GraphPad | http://www.graphpad.com/scientific-software/prism/ |
| Adobe Illustrator | Adobe | http://www.adobe.com/ |
| Adobe Photoshop | Adobe | http://www.adobe.com/ |
| ImageJ | NIH | http://imagej.nih.gov/ij/ |
| Zen 3.1 image processing software | Zeiss | http://www.zeiss.com/ |
| Leica Application Suite Advanced Fluorescence (LAS AF) Software | Leica | http://www.leica-microsystems.com/home/ |

**SUPPLEMENTAL FIGURES**

**
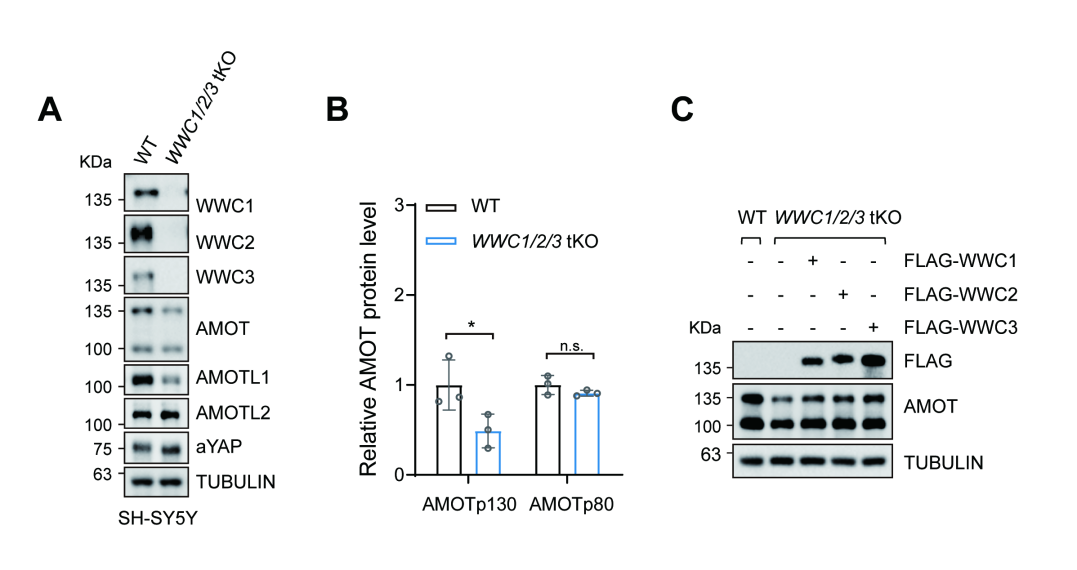
**

**Fig. S1 WWC1/2/3 regulate protein stability of Motins.** (A, B) Motins protein levels are decreased in *WWC1/2/3* tKO SH-SY5Y cells. Protein expression was determined by immunoblotting (A) and quantified (B). Data are shown as the mean ± SD of three independent experiments. **p* < 0.05, n.s. (not significant) between indicated groups. (C) Ectopic WWC rescues the expression of AMOTp130 in *WWC1/2/3* tKO HEK293A cells. Representative immunoblot shows expression of endogenous of AMOT in *WWC1/2/3* tKO HEK293A cells transfected with the indicated plasmids.

**
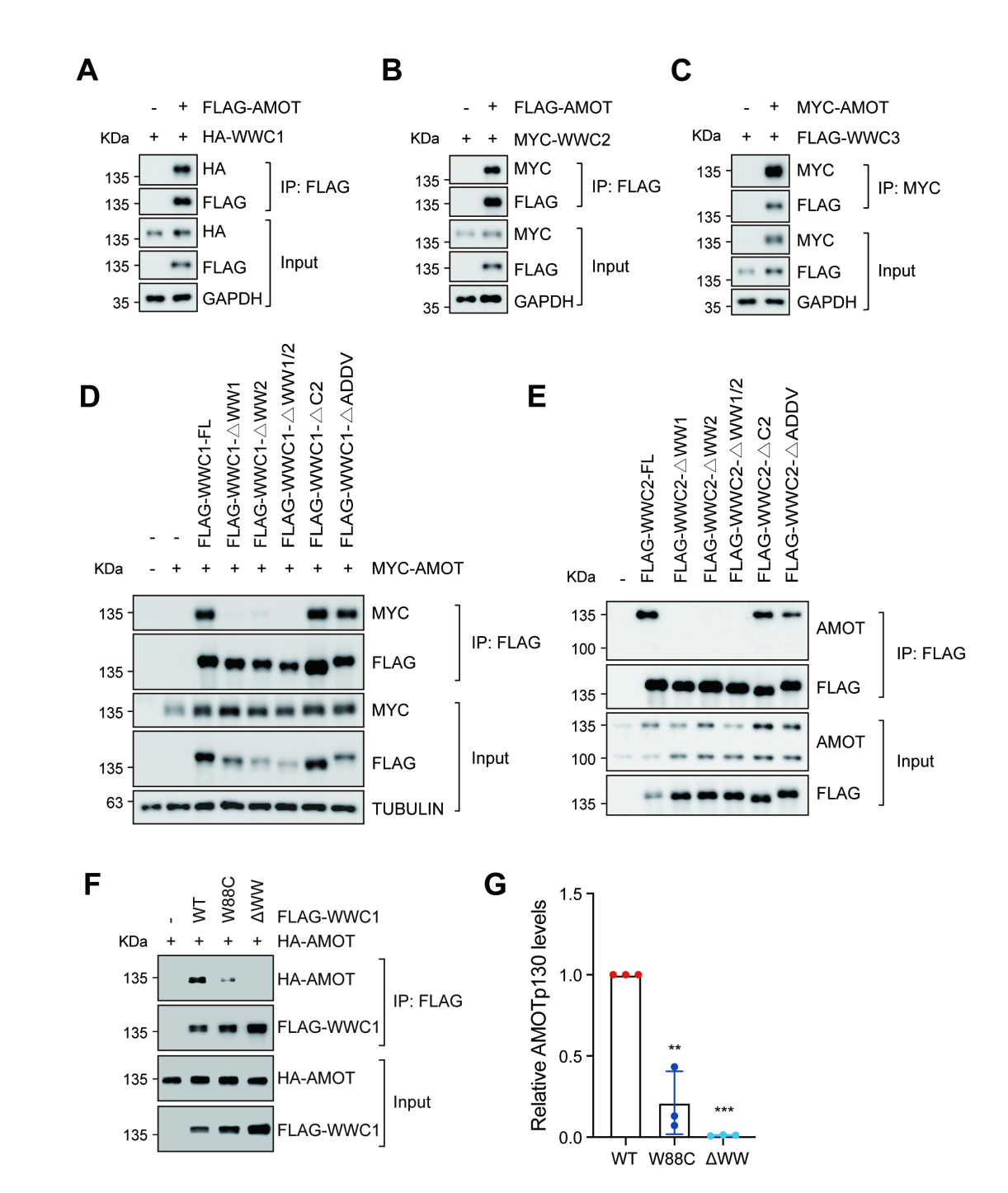
**

**Fig. S2 WWC proteins interact directly with and stabilize Motins.** (A-C) AMOTp130 interacts with WWC1 (A), WWC2 (B), and WWC3 (C). HEK293A cells were co-transfected with the indicated plasmids. Cell lysates were subjected to immunoprecipitation and immunoblotting. (D, E) C2-like domain and PDZ-binding domain in WWC1/2 are not required for interaction with AMOTp130. (D) WWC1 truncations and WT WWC1 (FLAG tagged) were expressed in HEK293A cells and immunoprecipitated with FLAG-beads. (E) WWC2 truncations and WT WWC2 (FLAG tagged) were expressed in HEK293A cells and immunoprecipitated with FLAG-beads. Bound AMOTp130 (MYC tagged or endogenous, D and E respectively) was analyzed by immunoblotting. (F, G) Patient-derived WWC1 mutant (W88C) binds to AMOTp130 weakly. WT WWC1, W88C-WWC1, △WW-WWC1 (FLAG tagged) and HA-AMOTp130 were co-expressed in HEK293A cells, and whole cell lysates were subjected to immunoprecipitation and immunoblotting. Quantification is shown in (G). Data are shown as the mean ± SD of three independent experiments; ***p* < 0.01, ****p* < 0.001 between indicated groups.


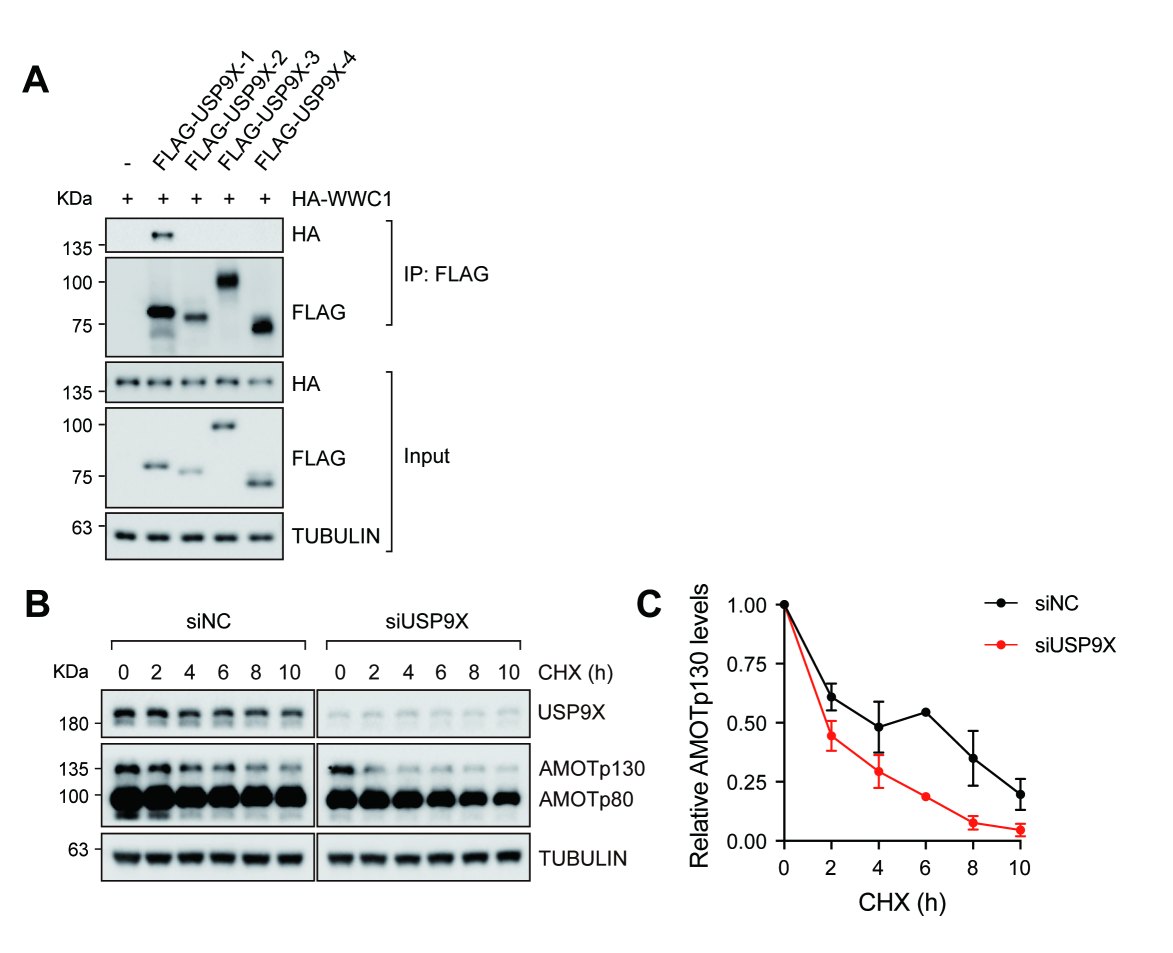


**Fig. S3 WWC proteins recruit USP9X to deubiquitinate and stabilize Motins.** (A) Interaction of USP9X mutants with WWC1. USP9X was truncated into four fragments (1: 1-600 aa; 2: 601-1200 aa; 3: 1201-2000 aa; 4: 2001-2555 aa). USP9X mutants (FLAG tagged) and HA-WWC1 were co-transfected into HEK293A cells. Cell lysates were subjected to immunoprecipitation and immunoblotting. (B, C) USP9X regulates protein stability of AMOTp130. (B) Representative immunoblot of endogenous AMOT in wild-type or *USP9X*-depleted HEK293A cells treated with cycloheximide (CHX; 100 mg/mL for 2 to 10 h). Quantification is shown in (C). Data are shown as the mean ± SD of three independent experiments.


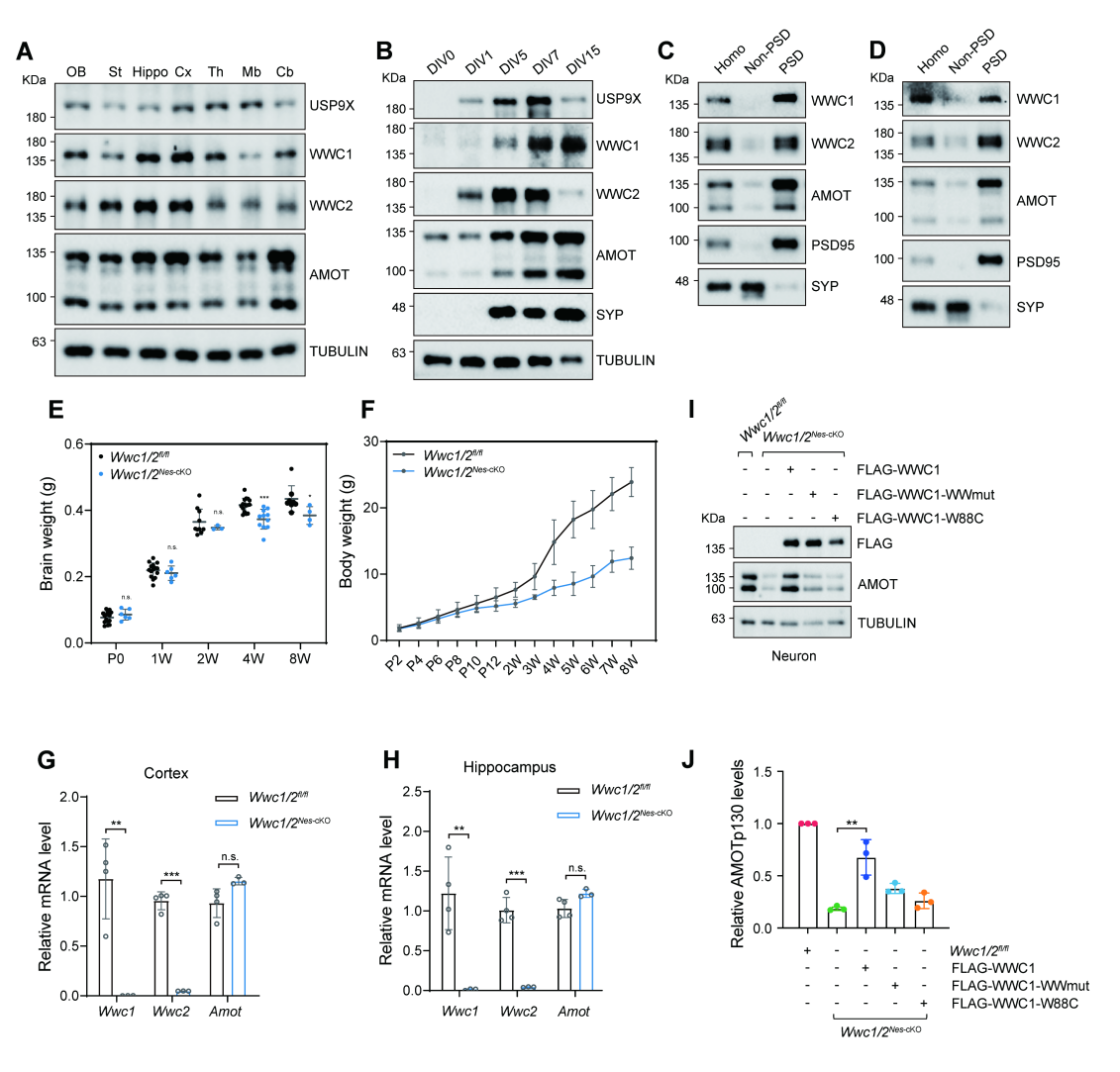


**Fig. S4 WWC proteins regulate stability of Motins in neurons and brain.** (A) Representative immunoblot analysis of WWC proteins expression in different brain areas. “OB”: olfactory bulbs; “St”: striatum; “Hippo”: hippocampus; “Cx”: cortex; “Th”: thalamus; “Mb”: midbrain; “Cb”: cerebellum. (B) WWC proteins expression increases during neuronal development *in vitro*. Cell lysates from primary cultured cortical neurons on the indicated days *in vitro* (DIV) were analyzed by immunoblotting. (C, D) Motins and WWC1/2 are enriched in PSD. Immunoblotting analysis of WWC proteins and Motins in postsynaptic density isolated from cortex (C) and hippocampus (D) are shown, respectively. “Homo” = without fractionation; “Non-PSD” = non postsynaptic density; “PSD” = postsynaptic density. (E) Smaller brain associates with *Wwc1/2* deletion. The whole brain weight of *Wwc1/2^fl/fl^* and *Wwc1/2^Nes^*^-cKO^ mice was evaluated at different ages. P0 to 8W, *Wwc1/2^fl/fl^*, n=15, 15, 10, 16, 8; P0 to 8W, *Wwc1/2^Nes^*^-cKO^, n=6, 6, 3, 11, 4. Data are shown as the mean ± SD; **p* < 0.05, ****p* < 0.001, and n.s. (not significant) between indicated groups. (F) Smaller body associates with *Wwc1/2* deletion. The body weight of *Wwc1/2^fl/fl^* (n=6) and *Wwc1/2^Nes^*^-cKO^ (n=5) mice was evaluated at different ages. Data are shown as the mean ± SD. (G, H) Validation of *Wwc1/2* knockout efficiency in cortex (G) and hippocampus (H) from *Wwc1/2^fl/fl^* (n=4) and *Wwc1/2^Nes^*^-cKO^ (n=3) mice was performed by qRT-PCR. Data are shown as the mean ± SD; ***p* < 0.01, ****p* < 0.001, and n.s. (not significant) between indicated groups. (I, J) Ectopic WT WWC1 but not WWC1 mutants can strongly rescue the expression of AMOTp130 in *Wwc1/2* knockout primary neurons. Protein expression was determined by immunoblotting (I) and quantified (J). Data are shown as the mean ± SD of three independent experiments; ***p* < 0.01 between indicated groups.


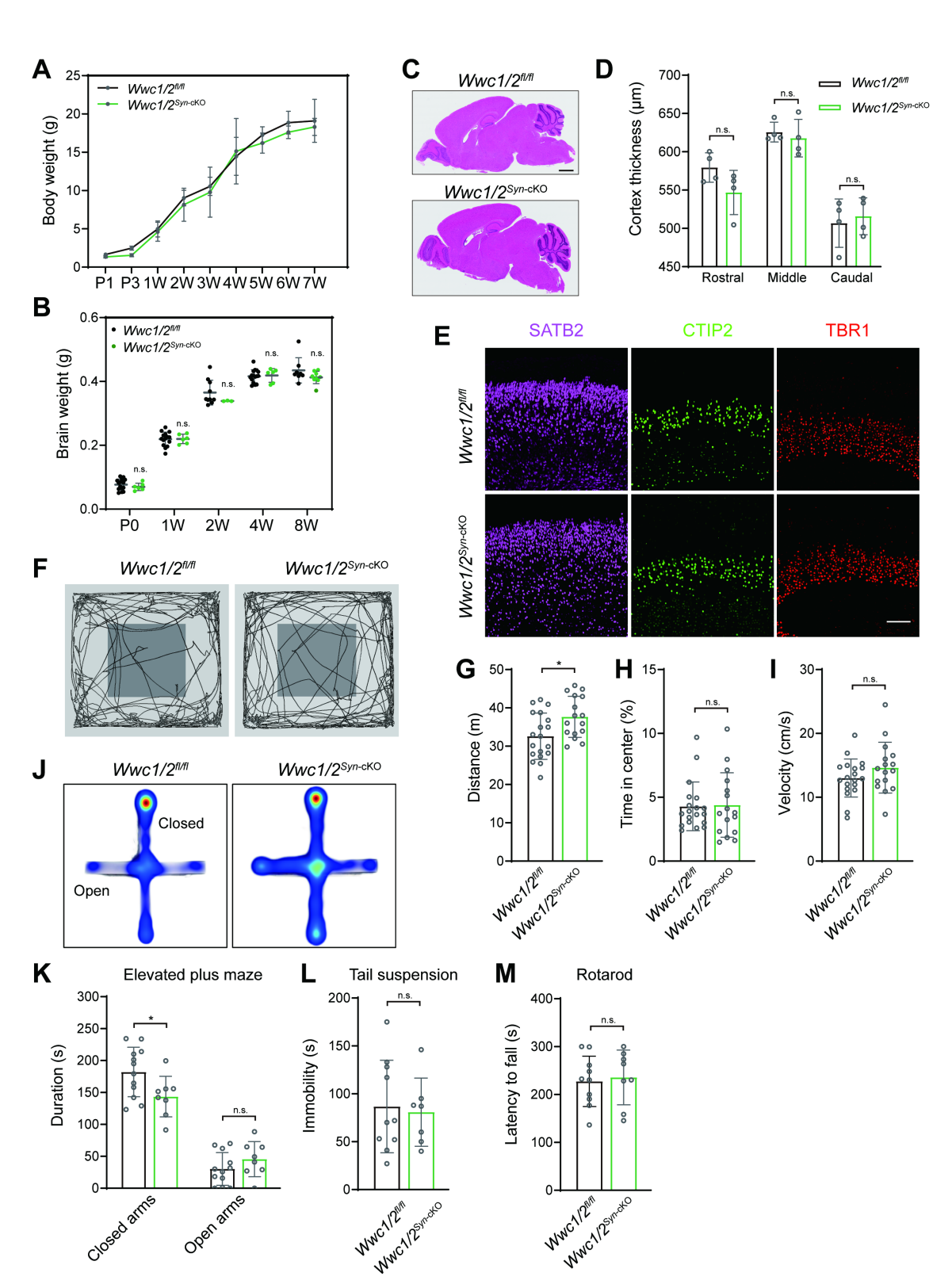


**Fig. S5 Defective spinogenesis and cognition in WWC1/2-deficient mice.** (A) The body weight of *Wwc1/2^fl/fl^* (n=5) and *Wwc1/2^Syn^*^-cKO^ (n=5) mice was evaluated at different ages. (B) The whole brain weight of *Wwc1/2^fl/fl^* and *Wwc1/2^Syn^*^-cKO^ mice was evaluated at different ages. P0 to 8W, *Wwc1/2^fl/fl^*, n=15, 15, 10, 16, 8; P0 to 8W, *Wwc1/2^Syn^*^-cKO^, n=6, 6, 3, 6, 9. Data are shown as the mean ± SD; n.s. (not significant) between indicated groups. (C) Hematoxylin-eosin staining of sagittal sections of the whole brain of *Wwc1/2^fl/fl^* and *Wwc1/2^Syn^*^-cKO^ mice at P21. Scale bar, 600 μm. (D) Quantification of thickness of neocortex for *Wwc1/2^fl/fl^* and *Wwc1/2^Syn^*^-cKO^ mice at P0 (n=3 per genotype) is shown. Data are shown as the mean ± SD; n.s. (not significant) between indicated groups. (E) Representative images of P0 *Wwc1/2^fl/fl^* and *Wwc1/2^Syn^*^-cKO^ cortices stained for SATB2, CTIP2 and TBR1. Scale bar, 25 μm. (F) Representative traces of *Wwc1/2^fl/fl^* mice and their *Wwc1/2^Syn^*^-cKO^ littermates in the open-field arena is shown. The shaded area represents central area in the open field. (G-I) Quantifications of the total distance (G), time in center (H), and velocity (I) in the open field test are shown. *Wwc1/2^fl/fl^*, n=19; *Wwc1/2^Syn^*^-cKO^, n=16. Data are shown as the mean ± SD; **p* < 0.05, n.s. (not significant) between indicated groups. (J) Representative tracing heatmap analysis of *Wwc1/2^fl/fl^* mice and their *Wwc1/2^Syn^*^-cKO^ littermates in elevated plus maze test is shown. (K) Quantification of total time spent in the closed arms and open arms of the elevated plus maze test is shown. *Wwc1/2^fl/fl^*, n=12; *Wwc1/2^Syn^*^-cKO^, n=8. Data are shown as the mean ± SD; **p* < 0.05, n.s. (not significant) between indicated groups. (L) Quantification of total immobile time in the tail suspension test is shown. *Wwc1/2^fl/fl^*, n=10; *Wwc1/2^Syn^*^-cKO^, n=7. Data are shown as the mean ± SD; n.s. (not significant) between indicated groups. (M) Quantification of the latency to fall in the rotarod test is shown. *Wwc1/2^fl/fl^*, n=10; *Wwc1/2^Syn^*^-cKO^ , n=8. Data are shown as the mean ± SD; n.s. (not significant) between indicated groups.


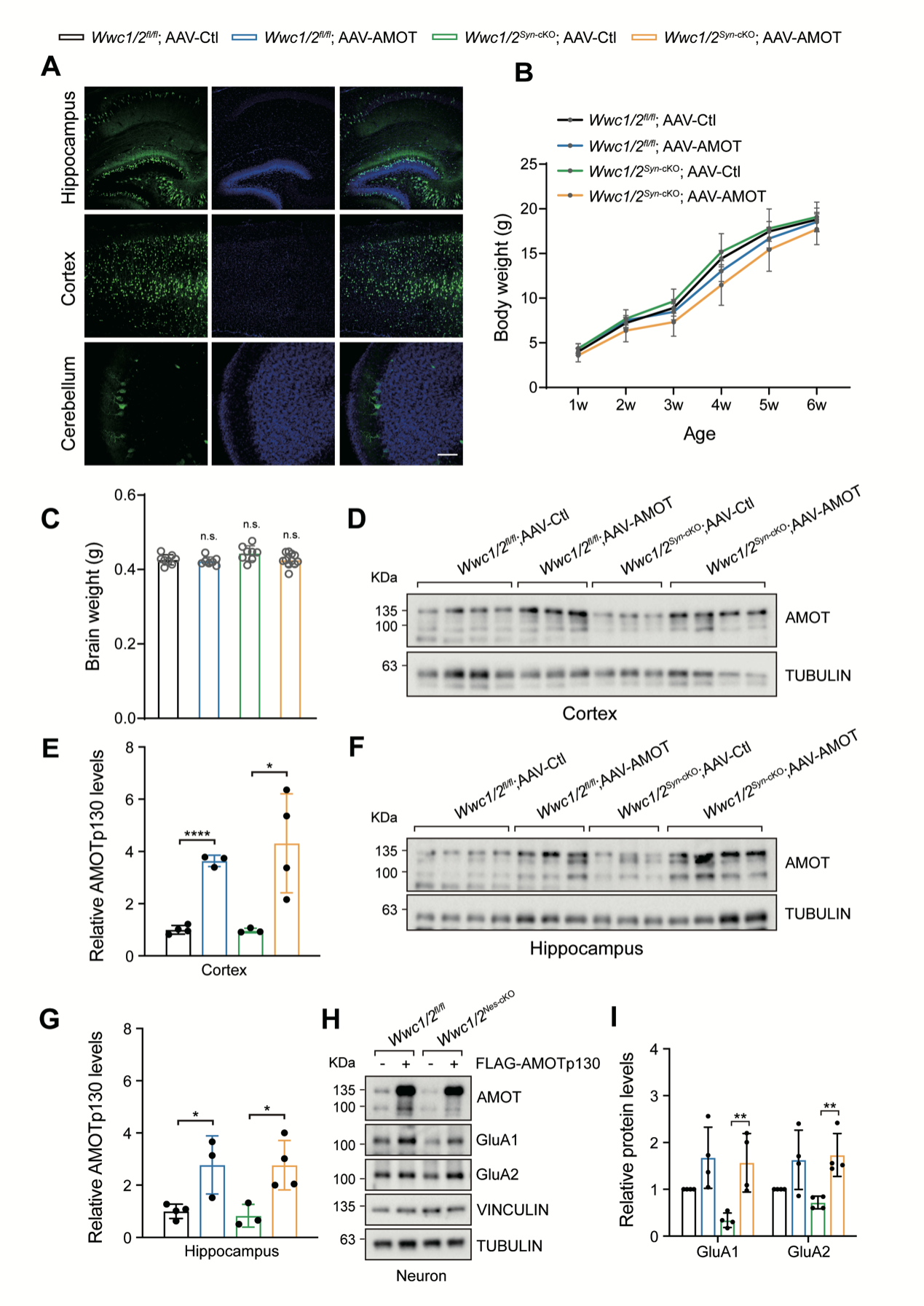


**Fig. S6 The phenotype of WWC1/2 loss is partially rescued by ectopic expression of AMOT.** (A) Representative images taken from mice harvested 2 weeks after injection with AAV2/9-Syn-ZsGreen (1.0 × 10^8 particles / hemisphere). Representative images of hippocampus (upper), cortex (middle) and cerebellum (lower) are shown. Viral transduction was visualized by native ZsGreen fluorescence. Scale bar, 100 μm. (B) The body weight of *Wwc1/2^fl/fl^* mice injected with AAV2/9-vector (n=6) or AAV2/9-AMOT (n=9); *Wwc1/2^Syn^*^-cKO^ mice injected with AAV2/9-vector (n=7) or AAV2/9-AMOT (n=12) was evaluated at different ages. Data are shown as the mean ± SD. (C) The brain weight of *Wwc1/2^fl/fl^* mice injected with AAV2/9-vector (n=8) or AAV2/9-AMOT (n=7); *Wwc1/2^Syn^*^-cKO^ mice injected with AAV2/9-vector (n=8) or AAV2/9-AMOT (n=12) was evaluated at 8W. Data are shown as the mean ± SD; n.s. (not significant) between indicated groups. (D-G) Validation of neuronal transduction two months after intracerebroventricular AAV2/9 injection. Immunoblotting analysis of the cortex (D) and hippocampus (F) was performed to detect endogenous protein level of AMOT. Quantifications are shown in (E, cortex) and (G, hippocampus). *Wwc1/2^fl/fl^* mice were injected with AAV2/9-vector (n=4) or AAV2/9-AMOT (n=3); *Wwc1/2^Syn^*^-cKO^ mice were injected with AAV2/9-vector (n=3) or AAV2/9-AMOT (n=4). Data are shown as the mean ± SD; **p* < 0.05, *****p* < 0.0001 between indicated groups. (H, I) AMOT involves in AMPAR regulation. Primary cortical neurons extracted from *Wwc1/2^fl/fl^* and *Wwc1/2^Nes^*^-cKO^ mice were infected with lentivirus-vector and/or lentivirus-AMOTp130. Cell lysates were subjected to immunoblotting analysis of GluA1 and GluA2 (H). Quantification is shown in (I). Data are shown as the mean ± SD of four independent experiments; **p* < 0.05, ***p* < 0.01 between indicated groups.
