# Supplementary material for: WWC1/2 regulate spinogenesis and cognition in mice by stabilizing AMOT
Source: Cell Death Dis. 2023 Aug 1;14(8):491. doi: 10.1038/s41419-023-06020-7 (PMC10394084; doi:10.1038/s41419-023-06020-7)
Supplement: Supplementary file 9 — Confirmation of authorship [file 41419_2023_6020_MOESM9_ESM.pdf]

Runyi Cao

主　题:	Re: Confirmation on authorship changes_CDDIS-22-5085RRR
发件人:	"曹润怿" <18111240002@fudan.edu.cn> 2023-7-18 11:26:22
收件人:	"yu, fa-xing 余发星" <fxyu@fudan.edu.cn>

Dear Professor Yu,

I agree to these changes.

Best wishes,

Runyi Cao

-----原始邮件-----
发件人:"yu, fa-xing 余发星" <fxyu@fudan.edu.cn>
发送时间:2023-07-18 10:46:40 (星期二)
收件人: 18111240002@fudan.edu.cn, r_zhu@fudan.edu.cn, 1286064525@qq.com, 17111510051@fudan.edu.cn, leiyubin@westlake.edu.cn, yf_tan@fudan.edu.cn, 18111510024@fudan.edu.cn, fd_wangyu@fudan.edu.cn, yiwang@shmu.edu.cn, zxzhong20@fudan.edu.cn, zhengfengyun@fudan.edu.cn
抄送: fxyu@fudan.edu.cn
主题: Confirmation on authorship changes_CDDIS-22-5085RRR

Dear coauthors,

Our manuscript has been accepted in principle at Cell Death & Disease. During two round revision, the manuscript has been greatly improved, with the help of additional investigators. We would like to add Drs. Yu Wang and Yi Wang as coauthors, to reflex their inputs in this work. However, these changes should be supported by all contributing authors. I have attached the latest version of cover page below for your reference. Please reply my email and let me know if you are agree to these changes. Thank you.

Best wishes,

Fa-Xing Yu

Rui Zhu

主　题:	Re: Confirmation on authorship changes_CDDIS-22-5085RRR	
发件人:	"朱锐" <r_zhu@fudan.edu.cn>	2023-7-18 11:06:18
收件人:	"yu, fa-xing 余发星" <fxyu@fudan.edu.cn>	

Agree with that.

---- Replied Message ----

From	yu, fa-xing 余发星<fxyu@fudan.edu.cn>
Date	07/18/2023 10:50
To	18111240002@fudan.edu.cn<18111240002@fudan.edu.cn>,<br>r_zhu@fudan.edu.cn<r_zhu@fudan.edu.cn>,<br>1286064525@qq.com<1286064525@qq.com>,<br>17111510051@fudan.edu.cn<17111510051@fudan.edu.cn>,<br>leiyubin@westlake.edu.cn<leiyubin@westlake.edu.cn>,<br>yf_tan@fudan.edu.cn<yf_tan@fudan.edu.cn>,<br>18111510024@fudan.edu.cn<18111510024@fudan.edu.cn>,<br>fd_wangyu@fudan.edu.cn<fd_wangyu@fudan.edu.cn>,<br>yiwang@shmu.edu.cn<yiwang@shmu.edu.cn>,<br>zxzhong20@fudan.edu.cn<zxzhong20@fudan.edu.cn>,<br>zhengfengyun@fudan.edu.cn<zhengfengyun@fudan.edu.cn>
Cc	fxyu@fudan.edu.cn<fxyu@fudan.edu.cn>
Subject	Confirmation on authorship changes_CDDIS-22-5085RRR

Dear coauthors,

Our manuscript has been accepted in principle at Cell Death & Disease. During two round revision, the manuscript has been greatly improved, with the help of additional investigators. We would like to add Drs. Yu Wang and Yi Wang as coauthors, to reflex their inputs in this work. However, these changes should be supported by all contributing authors. I have attached the latest version of cover page below for your reference. Please reply my email and let me know if you are agree to these changes. Thank you.

Best wishes,

Fa-Xing Yu

Zhao Sha

主　题:	Re: Confirmation on authorship changes_CDDIS-22-5085RRR	
发件人:	"沙钊" <1286064525@qq.com>	2023-7-18 11:11:13
收件人:	"yu, fa-xing 余发星" <fxyu@fudan.edu.cn>	

Dear Dr.Yu

I have reviewed the attached latest version of the cover page, and I agree with the proposed the addition.

Best regards,

---Original---

From: "yu, fa-xing 余发星"<fxyu@fudan.edu.cn>
Date: Tue, Jul 18, 2023 10:46 AM
To: "18111240002"<18111240002@fudan.edu.cn>;"r_zhu"<r_zhu@fudan.edu.cn>;"1286064525"<1286064525@qq.com>;"17111510051"<17111510051@fudan.edu.cn>;"leiyubin"<leiyubin@westlake.edu.cn>;"yf_tan"<yf_tan@fudan.edu.cn>;"18111510024"<18111510024@fudan.edu.cn>;"fd_wangyu"<fd_wangyu@fudan.edu.cn>;"yiwang"<yiwang@shmu.edu.cn>;"zxzhong20"<zxzhong20@fudan.edu.cn>;"zhengfengyun"<zhengfengyun@fudan.edu.cn>;
Cc: "fxyu"<fxyu@fudan.edu.cn>;
Subject: Confirmation on authorship changes_CDDIS-22-5085RRR

Dear coauthors,

Our manuscript has been accepted in principle at Cell Death & Disease. During two round revision, the manuscript has been greatly improved, with the help of additional investigators. We would like to add Drs. Yu Wang and Yi Wang as coauthors, to reflex their inputs in this work. However, these changes should be supported by all contributing authors. I have attached the latest version of cover page below for your reference. Please reply my email and let me know if you are agree to these changes. Thank you.

Best wishes,

Fa-Xing Yu

Sixian Qi

主　题:	回复：Confirmation on authorship changes_CDDIS-22-5085RRR
发件人:	"17111510051@fudan.edu.cn" <17111510051@fudan.edu.cn> 2023-7-18 13:38:21
收件人:	"Faxing YU" <fxyu@fudan.edu.cn>

Agree

17111510051

复旦大学
Fudan University

复旦大学
邮箱：17111510051@fudan.edu.cn

---- 回复的原邮件 ----

发件人	yu, fa-xing 余发星<fxyu@fudan.edu.cn>
日期	2023年07月18日 10:46
收件人	18111240002@fudan.edu.cn、r_zhu@fudan.edu.cn、1286064525@qq.com、17111510051@fudan.edu.cn、leiyubin@westlake.edu.cn、yf_tan@fudan.edu.cn、18111510024@fudan.edu.cn、fd_wangyu@fudan.edu.cn、yiwang@shmu.edu.cn、zxzhong20@fudan.edu.cn、zhengfengyun@fudan.edu.cn
抄送至	fxyu@fudan.edu.cn
主题	Confirmation on authorship changes_CDDIS-22-5085RRR

Dear coauthors,

Our manuscript has been accepted in principle at Cell Death & Disease. During two round revision, the manuscript has been greatly improved, with the help of additional investigators. We would like to add Drs. Yu Wang and Yi Wang as coauthors, to reflex their inputs in this work. However, these changes should be supported by all contributing authors. I have attached the latest version of cover page below for your reference. Please reply my email and let me know if you are agree to these changes. Thank you.

Best wishes,

Fa-Xing Yu

Zhenxing Zhong

主　题:	Re: Confirmation on authorship changes_CDDIS-22-5085RRR
发件人:	"钟振兴" <zxzhong20@fudan.edu.cn> 2023-7-18 11:19:21
收件人:	"\"yu, fa-xing 余发星\"" <fxyu@fudan.edu.cn>
附　件:	desc.png<br>ATT00003.txt

I agree with these changes.

Zhenxing Zhong

钟振兴
> 在 2023年7月18日，10:50，yu, fa-xing 余发星 <fxyu@fudan.edu.cn> 写道：
>
> Dear coauthors,
>
> Our manuscript has been accepted in principle at Cell Death & Disease. During two round revision, the manuscript has been greatly improved, with the help of additional investigators. We would like to add Drs. Yu Wang and Yi Wang as coauthors, to reflex their inputs in this work. However, these changes should be supported by all contributing authors. I have attached the latest version of cover page below for your reference. Please reply my email and let me know if you are agree to these changes. Thank you.
>
>
> Best wishes,
>
> Fa-Xing Yu
>
>

Fengyun Zheng

主　题:	Re: Confirmation on authorship changes_CDDIS-22-5085RRR
发件人:	zhengfengyun <zhengfengyun@fudan.edu.cn> 2023-7-18 14:15:56
收件人:	"yu, fa-xing 余发星" <fxyu@fudan.edu.cn>

Dear Professor Yu,

I agree to these changes.

Fengyun Zheng

-----原始邮件-----
发件人:"yu, fa-xing 余发星" <fxyu@fudan.edu.cn>
发送时间:2023-07-18 10:46:40 (星期二)
收件人: 18111240002@fudan.edu.cn, r_zhu@fudan.edu.cn, 1286064525@qq.com, 17111510051@fudan.edu.cn, leiyubin@westlake.edu.cn, yf_tan@fudan.edu.cn, 18111510024@fudan.edu.cn, fd_wangyu@fudan.edu.cn, yiwang@shmu.edu.cn, zxzhong20@fudan.edu.cn, zhengfengyun@fudan.edu.cn
抄送: fxyu@fudan.edu.cn
主题: Confirmation on authorship changes_CDDIS-22-5085RRR

Dear coauthors,

Our manuscript has been accepted in principle at Cell Death & Disease. During two round revision, the manuscript has been greatly improved, with the help of additional investigators. We would like to add Drs. Yu Wang and Yi Wang as coauthors, to reflex their inputs in this work. However, these changes should be supported by all contributing authors. I have attached the latest version of cover page below for your reference. Please reply my email and let me know if you are agree to these changes. Thank you.

Best wishes,

Fa-Xing Yu

Yubin Lei

主　题:	答复: Confirmation on authorship changes_CDDIS-22-5085RRR
发件人:	"Yubin LEI 雷雨斌" <leiyubin@westlake.edu.cn> 2023-7-18 11:39:31
收件人:	"yu, fa-xing 余发星" <fxyu@fudan.edu.cn>

I Agree!

____

发件人: yu, fa-xing 余发星 <fxyu@fudan.edu.cn>
发送时间: 2023年7月18日 10:46:40
收件人: 18111240002@fudan.edu.cn <18111240002@fudan.edu.cn>; r_zhu@fudan.edu.cn <r_zhu@fudan.edu.cn>; 1286064525@qq.com <1286064525@qq.com>; 17111510051@fudan.edu.cn <17111510051@fudan.edu.cn>; Yubin LEI 雷雨斌 <leiyubin@westlake.edu.cn>; yf_tan@fudan.edu.cn <yf_tan@fudan.edu.cn>; 18111510024@fudan.edu.cn <18111510024@fudan.edu.cn>; fd_wangyu@fudan.edu.cn <fd_wangyu@fudan.edu.cn>; yiwang@shmu.edu.cn <yiwang@shmu.edu.cn>; zxzhong20@fudan.edu.cn <zxzhong20@fudan.edu.cn>; zhengfengyun@fudan.edu.cn <zhengfengyun@fudan.edu.cn>
抄送: fxyu@fudan.edu.cn <fxyu@fudan.edu.cn>
主题: Confirmation on authorship changes_CDDIS-22-5085RRR

Dear coauthors,

Our manuscript has been accepted in principle at Cell Death & Disease. During two round revision, the manuscript has been greatly improved, with the help of additional investigators. We would like to add Drs. Yu Wang and Yi Wang as coauthors, to reflex their inputs in this work. However, these changes should be supported by all contributing authors. I have attached the latest version of cover page below for your reference. Please reply my email and let me know if you are agree to these changes. Thank you.

Best wishes,

Fa-Xing Yu

[cid:10513d2d$1$18966e48820$Coremail$fxyu$fudan.edu.cn]

Yanfeng Tan

主　题:	Re: Confirmation on authorship changes_CDDIS-22-5085RRR	
发件人:	YanfengTan <yf_tan@fudan.edu.cn>	2023-7-18 11:45:27
收件人:	"yu, fa-xing 余发星" <fxyu@fudan.edu.cn>	

Dear Professor Yu,
I agree to these changes.
Yanfeng Tan

-----原始邮件-----
发件人:"yu, fa-xing 余发星" <fxyu@fudan.edu.cn>
发送时间:2023-07-18 10:46:40 (星期二)
收件人: 18111240002@fudan.edu.cn, r_zhu@fudan.edu.cn, 1286064525@qq.com, 17111510051@fudan.edu.cn, leiyubin@westlake.edu.cn, yf_tan@fudan.edu.cn, 18111510024@fudan.edu.cn, fd_wangyu@fudan.edu.cn, yiwang@shmu.edu.cn, zxzhong20@fudan.edu.cn, zhengfengyun@fudan.edu.cn
抄送: fxyu@fudan.edu.cn
主题: Confirmation on authorship changes_CDDIS-22-5085RRR

Dear coauthors,

Our manuscript has been accepted in principle at Cell Death & Disease. During two round revision, the manuscript has been greatly improved, with the help of additional investigators. We would like to add Drs. Yu Wang and Yi Wang as coauthors, to reflex their inputs in this work. However, these changes should be supported by all contributing authors. I have attached the latest version of cover page below for your reference. Please reply my email and let me know if you are agree to these changes. Thank you.

Best wishes,

Fa-Xing Yu

Yuwen Zhu

主　题:	Re: Confirmation on authorship changes_CDDIS-22-5085RRR	
发件人:	18111510024 <18111510024@fudan.edu.cn>	2023-7-18 13:36:45
收件人:	"Faxing YU" <fxyu@fudan.edu.cn>	

I agree to these changes，thank you.

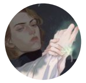

18111510024

邮箱：18111510024@fudan.edu.cn

---- Replied Message ----

From	yu, fa-xing 余发星<fxyu@fudan.edu.cn>
Date	07/18/2023 10:49
To	18111240002@fudan.edu.cn<18111240002@fudan.edu.cn>, r_zhu@fudan.edu.cn<r_zhu@fudan.edu.cn>, 1286064525@qq.com<1286064525@qq.com>, 17111510051@fudan.edu.cn<17111510051@fudan.edu.cn>, leiyubin@westlake.edu.cn<leiyubin@westlake.edu.cn>, yf_tan@fudan.edu.cn<yf_tan@fudan.edu.cn>, 18111510024@fudan.edu.cn<18111510024@fudan.edu.cn>, fd_wangyu@fudan.edu.cn<fd_wangyu@fudan.edu.cn>, yiwang@shmu.edu.cn<yiwang@shmu.edu.cn>, zxzhong20@fudan.edu.cn<zxzhong20@fudan.edu.cn>, zhengfengyun@fudan.edu.cn<zhengfengyun@fudan.edu.cn>
Cc	fxyu@fudan.edu.cn<fxyu@fudan.edu.cn>
Subject	Confirmation on authorship changes_CDDIS-22-5085RRR

Dear coauthors,

Our manuscript has been accepted in principle at Cell Death & Disease. During two round revision, the manuscript has been greatly improved, with the help of additional investigators. We would like to add Drs. Yu Wang and Yi Wang as coauthors, to reflex their inputs in this work. However, these changes should be supported by all contributing authors. I have attached the latest version of cover page below for your reference. Please reply my email and let me know if you are agree to these changes. Thank you.

Best wishes,

Yu Wang

主　题:	Re: Confirmation on authorship changes_CDDIS-22-5085RRR
发件人:	"YU WANG" <fd_wangyu@fudan.edu.cn> 2023-7-18 10:57:39
收件人:	"\"yu, fa-xing 余发星\"" <fxyu@fudan.edu.cn>

Dear Dr.Yu

I have reviewed the attached latest version of the cover page, and I agree with the proposed addition of Drs. Yu Wang and Yi Wang as coauthors

Best regards,

Yu Wang

> 2023年7月18日 10:46, yu, fa-xing 余发星 <fxyu@fudan.edu.cn> 写道:
>
> Dear coauthors,
>
> Our manuscript has been accepted in principle at Cell Death & Disease. During two round revision, the manuscript has been greatly improved, with the help of additional investigators. We would like to add Drs. Yu Wang and Yi Wang as coauthors, to reflex their inputs in this work. However, these changes should be supported by all contributing authors. I have attached the latest version of cover page below for your reference. Please reply my email and let me know if you are agree to these changes. Thank you.
>
>
> Best wishes,
>
> Fa-Xing Yu
>
>
> <desc.png>
>
>
>

Yi Wang

主　题:	Re: Confirmation on authorship changes_CDDIS-22-5085RRR
发件人:	"王艺" <yiwang@shmu.edu.cn> 2023-7-18 11:08:59
收件人:	"yu, fa-xing 余发星" <fxyu@fudan.edu.cn>

I agree to these changes.Thank you.
Yi

-----原始邮件-----
发件人:"yu, fa-xing 余发星" <fxyu@fudan.edu.cn>
发送时间:2023-07-18 10:46:40 (星期二)
收件人: 18111240002@fudan.edu.cn, r_zhu@fudan.edu.cn, 1286064525@qq.com, 17111510051@fudan.edu.cn, leiyubin@westlake.edu.cn, yf_tan@fudan.edu.cn, 18111510024@fudan.edu.cn, fd_wangyu@fudan.edu.cn, yiwang@shmu.edu.cn, zxzhong20@fudan.edu.cn, zhengfengyun@fudan.edu.cn
抄送: fxyu@fudan.edu.cn
主题: Confirmation on authorship changes_CDDIS-22-5085RRR

Dear coauthors,

Our manuscript has been accepted in principle at Cell Death & Disease. During two round revision, the manuscript has been greatly improved, with the help of additional investigators. We would like to add Drs. Yu Wang and Yi Wang as coauthors, to reflex their inputs in this work. However, these changes should be supported by all contributing authors. I have attached the latest version of cover page below for your reference. Please reply my email and let me know if you are agree to these changes. Thank you.

Best wishes,

Fa-Xing Yu

Fa-Xing Yu

July 18, 2023

Dear Editors,

As corresponding author of the manuscript (CDDIS-22-5085RRR), I agree to the changes on authorship made during revisions.

Best regards,

Fa-Xing Yu
Fudan University
